# Cellular Dynamics and Genomic Identity of Centromeres in Cereal Blast Fungus

**DOI:** 10.1128/mBio.01581-19

**Published:** 2019-07-30

**Authors:** Vikas Yadav, Fan Yang, Md. Hashim Reza, Sanzhen Liu, Barbara Valent, Kaustuv Sanyal, Naweed I. Naqvi

**Affiliations:** a Molecular Biology and Genetics Unit, Jawaharlal Nehru Centre for Advanced Scientific Research, Jakkur, Bangalore, India; b Temasek Life Sciences Laboratory, and the Department of Biological Sciences, National University of Singapore, Singapore; c Department of Plant Pathology, Kansas State University, Manhattan, Kansas, USA; University of Melbourne

**Keywords:** CenpA, centromeres, rice blast, wheat blast, chromosome structure, kinetochore dynamics

## Abstract

Precise kinetochore-microtubule interactions ensure faithful chromosome segregation in eukaryotes. Centromeres, identified as scaffolding sites for kinetochore assembly, are among the most rapidly evolving chromosomal loci in terms of the DNA sequence and length and organization of intrinsic elements. Neither the centromere structure nor the kinetochore dynamics is well studied in plant-pathogenic fungi. Here, we sought to understand the process of chromosome segregation in the rice blast fungus Magnaporthe oryzae. High-resolution imaging of green fluorescent protein (GFP)-tagged inner kinetochore proteins CenpA and CenpC revealed unusual albeit transient declustering of centromeres just before anaphase separation of chromosomes in M. oryzae. Strikingly, the declustered centromeres positioned randomly at the spindle midzone without an apparent metaphase plate *per se*. Using CenpA chromatin immunoprecipitation followed by deep sequencing, all seven centromeres in M. oryzae were found to be regional, spanning 57-kb to 109-kb transcriptionally poor regions. Highly AT-rich and heavily methylated DNA sequences were the only common defining features of all the centromeres in rice blast. Lack of centromere-specific DNA sequence motifs or repetitive elements suggests an epigenetic specification of centromere function in M. oryzae. PacBio genome assemblies and synteny analyses facilitated comparison of the centromeric/pericentromeric regions in distinct isolates of rice blast and wheat blast and in Magnaporthiopsis poae. Overall, this study revealed unusual centromere dynamics and precisely identified the centromere loci in the top model fungal pathogens that belong to *Magnaporthales* and cause severe losses in the global production of food crops and turf grasses.

## Abstract (precis)

Magnaporthe oryzae is an important fungal pathogen that causes a loss of 10% to 30% of the annual rice crop due to the devastating blast disease. In most organisms, kinetochores are clustered together or arranged at the metaphase plate to facilitate synchronized anaphase separation of sister chromatids in mitosis. In this study, we showed that the initially clustered kinetochores separate and position randomly prior to anaphase in M. oryzae. Centromeres in M. oryzae occupy large genomic regions and form on AT-rich DNA without any common sequence motifs. Overall, this study identified atypical kinetochore dynamics and mapped functional centromeres in M. oryzae to define the roles of centromeric and pericentric boundaries in kinetochore assembly on epigenetically specified centromere loci. This study should pave the way for further understanding of the contribution of heterochromatin in genome stability and virulence of the blast fungus and its related species of high economic importance.

## INTRODUCTION

Faithful chromosome segregation is an essential process required for maintaining genome integrity in dividing cells. This process is successfully carried out by the attachment of microtubules, emanating from opposite spindle poles, to the kinetochore, which is the proteinaceous multisubunit structure that is preassembled onto centromeres (1, 2). The centromere forms a crucial part of this machinery, and yet it is one of the most rapidly evolving loci in eukaryotic genomes (3, 4). In contrast, the proteins that bind to centromere DNA are evolutionarily conserved (2). Centromere DNA shows a wide diversity with respect to the length, composition, and organization of elements of the underlying DNA sequence. A few budding yeast species, such as Saccharomyces cerevisiae, harbor centromeres that are less than 400 bp, consisting of conserved DNA sequence elements that form point centromeres (5). Most other organisms possess regional centromeres that span a range of lengths from a few kilobases to several megabases. Unlike point centromeres, various epigenetic factors other than the DNA sequence determine the location and function of regional centromeres. For example, the regional centromeres in Schizosaccharomyces pombe and Candida tropicalis have a homogenized central core flanked by inverted repeats (6, 7). Likewise, the regional centromeres in Cryptococcus neoformans possess specific retrotransposons that are present randomly therein (8). In contrast, each of the centromeres in Candida albicans, Candida lusitaniae, and Candida dubliniensis is unique and thus believed to be epigenetically regulated (9–11). Indeed, several lines of evidence suggest that factors other than the DNA sequence *per se* are the determinants of centromeres in C. albicans (4, 12). These factors include the presence of preexisting molecules of centromeric histone Cse4 (CENP-A in humans) (13); clustering of centromeres to form a nuclear subdomain rich in centromeric histone (14); cross talk between DNA replication, DNA repair, and chromosome segregation machinery (15); and differential *cis* and *trans* interactions at the centromeres and pericentric regions (16). Centromeres in filamentous fungi such as Neurospora crassa, on the other hand, span long stretches of repetitive DNA but lack a consensus sequence or pattern (17, 18). Metazoans and plants also have regional centromeres that are a few megabases long and mostly consist of repetitive DNA or transposons (19–21). Centromeres in humans are composed of 170-bp α-satellite repeat sequences, which are further organized into higher-order repeats along the length of the centromeres (22). Centromeres in *Drosophila* are mainly composed of short repeat sequences, which are interspersed with transposons (23). Similarly, most centromeres in plants also contain retrotransposons and distinct satellite DNA sequences, with some being centromere specific (19, 24). For example, centromeres in maize are composed of the centromere-specific retrotransposon (CRM) elements (25). Unfortunately, repeat-rich centromere DNA sequences in most metazoans and plants remain poorly studied due to incomplete genome assembly spanning these regions.

Despite sequence divergence, centromeres in most studied organisms are bound by the centromere-specific histone H3 variant CENP-A/CenH3/Cse4, also known as the hallmark of centromere identity (4, 26). CENP-A forms the foundation of the kinetochore assembly and is essential for cell viability in all organisms studied to date. Evolutionary conservation of CENP-A along with other kinetochore proteins also provides an efficient tool to identify centromeres. Additionally, studies with fluorescently labeled inner kinetochore proteins such as CENP-A or CENP-C/Cen-C/Mif2 have led to an understanding of spatial dynamics of the kinetochore within the nucleus (27–31). Those studies established that the kinetochores in most yeast species are clustered throughout the nuclear division and, unlike metazoan centromeres, do not align on a metaphase plate. However, more recently, some variations in the metaphase plate or in kinetochore clustering have been reported, revealing the diversity in this phenomenon. Kinetochores were shown to remain clustered throughout the cell cycle in two well-studied ascomycetes, S. cerevisiae and C. albicans (32, 33). In S. pombe, kinetochores undergo a brief declustering during mitosis but remain clustered otherwise (27, 34). Another ascomycete, Zymoseptoria tritici, shows multiple kinetochore foci instead of a single cluster during interphase, although their localization dynamics during mitosis remains unexplored (35). On the other hand, the cells of a basidiomycete, C. neoformans, display multiple foci of kinetochores in interphase but the kinetochores gradually cluster during mitosis (28, 31). Even the phenomenon of centromere/kinetochore clustering that has been observed in *Drosophila* depends on centric chromatin rather than specific DNA sequences (36). Clustered centromeres are often localized near the nuclear periphery close to the spindle pole bodies (SPBs) in fungal species. Tethering of clustered centromeres to the nuclear envelope (NE) provides the so called Rabl conformation of chromosomes. Indeed, mutants affecting the tethering of chromosomes to the NE displayed aberrant chromosome segregation (31, 34, 37). Thus, clustering of centromeres may play a significant role in chromosome dynamics and in their timely separation during mitosis.

In addition to CENP-A, several other chromatin features are known to be associated with centromeres. For example, centromeres are devoid of genes and open reading frames (ORFs) and exhibit a significantly low level of poly(A) transcription compared to the rest of the genome (8, 38). Peripheral localization of centromeres in the nucleus, away from the active transcription zone, probably facilitates low levels of transcription from centromeres. Furthermore, centromeres in many organisms are heterochromatic in nature and harbor heterochromatic marks such as H3K9di/trimethylation and DNA methylation (8, 18, 39). A preference for AT-rich DNA sequence is evident for centromere formation in some organisms (18, 40–42). Note that none of these features exclusively define centromeres and that, in most cases, the importance of an individual factor in defining centromere loci is not well understood. However, the presence of such features on discrete chromosomal loci may pave the way for predicting centromeres in organisms in which genome tractability is difficult.

*Magnaporthales* is an order of ascomycete fungi comprising of many important plant-pathogenic species, including Magnaporthe oryzae (synonym of Pyricularia oryzae) and Magnaporthiopsis poae (43). M. oryzae includes host-adapted lineages (pathotypes) that cause the devastating blast diseases in cereal crops, including rice, wheat, barley, and millets (44–46). M. poae is responsible for summer-patch disease in turf grasses (47). The M. oryzae *Oryza* lineage causes rice blast, which remains a constant threat to agriculture-based economies due to significant damage to rice harvests. Recently, wheat blast disease, caused by the M. oryzae *Triticum* lineage, has emerged as a major threat to global wheat production (48). Rice blast has also become a model pathosystem for studying host-pathogen interactions due to the availability of the genome sequences, fully characterized infection cycle, genetic tractability, and economic significance of the fungus (49, 50). However, even with the availability of the genome sequence and annotated assembly, the centromere/kinetochore identity of the blast fungus remains unexplored or poorly defined. Here, we first studied and characterized orthologs of CENP-A and CENP-C, two well-conserved kinetochore proteins, to understand the kinetochore dynamics in the blast fungus and used these kinetochore proteins as tools to identify *bona fide* centromeres. Comparative analyses of *CEN* sequences were then carried out in diverse isolates of rice blast, wheat blast, and the summer patch pathogen belonging to the order *Magnaporthales*.

## RESULTS

### Kinetochores are clustered during interphase in M. oryzae.

**FIG 1:**
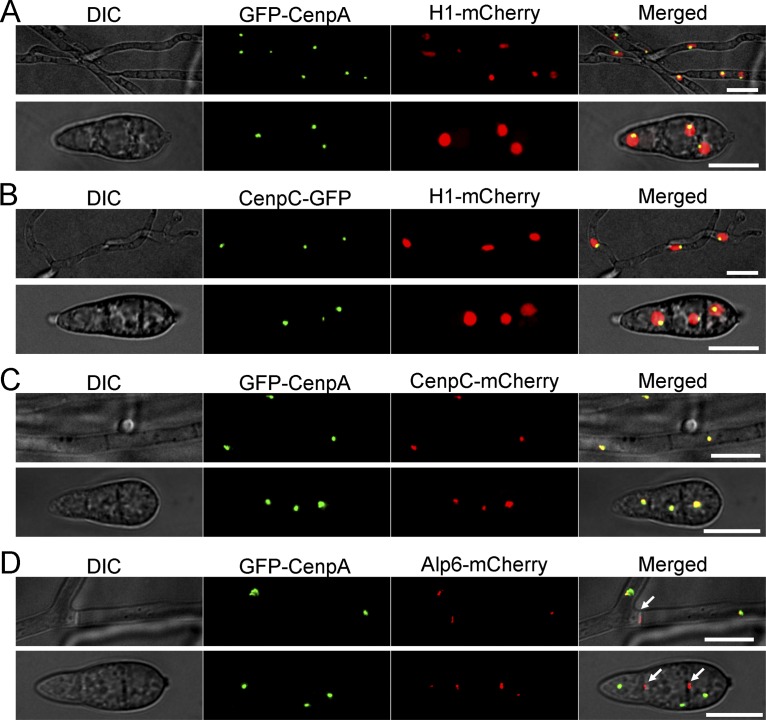
Localization patterns of CenpA and CenpC reveal that kinetochores are closely associated with each other in M. oryzae. (A) M. oryzae strain MGYF03 exhibited a single dot-like GFP-CenpA signal localized at the periphery of each nucleus marked by mCherry-histone H1 in both mycelia (upper panel) and conidia (lower panel). (B) Similarly, another inner kinetochore protein, CenpC-GFP in strain MGYF04, was found to be localized at the periphery of the mCherry-histone H1-marked nucleus in both mycelia (upper panel) and conidia (lower panel). (C) Colocalization of GFP-CenpA and CenpC-mCherry revealed complete overlapping signals in both mycelia and conidia in the MGYF05 strain. (D) In strain MGYF08, the clusters of GFP-CenpA were found to be closely associated with the spindle pole body (SPB) component Alp6-mCherry. In addition to SPBs, the Alp6 signals were also observed at the septa (white arrows). The fluorescence images shown here are maximum projections from Z stacks consisting of 0.5-μm-spaced planes. Bar, 10 μm. DIC, differential interference contrast.

A subset of putative kinetochore proteins was previously annotated in M. oryzae (17). We expanded the list further by identifying the putative orthologs of the additional conserved kinetochore proteins using *in silico* predictions and through multiple-sequence alignment established the identity of the two most conserved inner kinetochore proteins: CenpA (MGG_06445, an ortholog of CENP-A) and CenpC (MGG_06960, orthologous to CENP-C) (see Fig. S1 in the supplemental material) in M. oryzae. CenpA and CenpC of M. oryzae share 73% and 42% sequence identity with their N. crassa counterparts (CenH3 and CEN-C, respectively). Next, we functionally expressed green fluorescent protein (GFP)-tagged CenpA and CenpC from their native genomic loci in the wild-type Guy11 strain of M. oryzae. The GFP-CenpA and CenpC-GFP signals were single dot-like and colocalized on chromatin, marked by mCherry-tagged histone H1 (Fig. 1A and B). Further, colocalization of CenpA and CenpC signals confirmed their overlapping spatial positions in both mycelia and conidia (Fig. 1C). Clustering of kinetochores is a hallmark feature of many yeast and fungal genera. Such clustered kinetochores are often found in close proximity to the spindle pole bodies (SPBs) (51). We localized SPBs by tagging Alp6 (MGG_01815, an ortholog of S. cerevisiae Spc98) with mCherry and observed that SPBs localized close to the clustered GFP-CenpA signal in M. oryzae (Fig. 1D). Alp6-mCherry was also present at the septa in both mycelia and conidia (arrows in Fig. 1D). However, the Alp6-mCherry signal was more prominent at the septal pores in the conidia than the kinetochore signal. This indicates differential patterns of localization of Alp6 during the conidial and mycelial stages of development. Such an Alp6 localization pattern has also been observed in S. pombe and Aspergillus nidulans (52, 53). These results indicate that the kinetochore localization that occurs during interphase in M. oryzae is similar to that observed in other ascomycetes. Our repeated attempts to delete *CENPA* or *CENPC* in M. oryzae failed, indicating that both are essential for cell viability. This result was further corroborated by conditional repression of *CENPA* using the Tet-off system. The *Tet-GFP-CENPA* strain ceased to grow on culture media supplemented with doxycycline, a condition under which Tet-driven *CENPA* expression was shut down (Fig. S2A). Overall, the conserved sequence features and the subcellular localization patterns confirmed that CenpA and CenpC are evolutionarily conserved kinetochore proteins in M. oryzae. We infer that kinetochores remained clustered together adjacent to the SPBs during interphase in the blast fungus.

10.1128/mBio.01581-19.2 FIG S1 Identification of CenpA and CenpC in M. oryzae. (A) A schematic representation of the kinetochore complex. For simplification, only CenpA and CenpC are highlighted. (B) Alignment of CenpA protein sequence from M. oryzae (Mo) with those of Drosophila melanogaster (Dm), Mus musculus (Mm), Homo sapiens (Hs), Saccharomyces cerevisiae (Sc), Ustilago maydis (Um), Candida albicans (Ca), Cryptococcus neoformans (Cn), Schizosaccharomyces pombe (Sp), and Neurospora crassa (Nc). The C-terminal region of CenpA is an evolutionarily conserved histone-fold domain (HFD), whereas the N terminus exhibits a high level of sequence divergence. (C) Multiple-sequence alignment of CenpC protein sequence from M. oryzae (Mo) with other species. CenpC protein sequences revealed conservation of the CenpC box and the DNA binding “Cupin” domain. Download FIG S1, TIF file, 2.8 MB. Copyright © 2019 Yadav et al. 2019 Yadav et al. This content is distributed under the terms of the Creative Commons Attribution 4.0 International license.

10.1128/mBio.01581-19.3 FIG S2 CenpA is essential for cell growth and viability in M. oryzae. (A) Growth characteristics and colony morphology of strain MGYF01 and the wild-type M. oryzae Guy11 strain in the absence (−Dox) or presence (+Dox) of doxycycline (5 mg/liter). (B) Epifluorescence confocal imaging of strain MGYF06 showing the organization of nuclei (H1-mCherry), kinetochores (GFP-CenpA), and microtubules (GFP-TubA). In interphase, the microtubule signals are localized in the cytoplasm, whereas a single clustered dot of GFP-CenpA colocalizes with chromatin marker H1-mCherry in mycelia as well as conidia. (C) Epifluorescence confocal imaging of strain MGYF09 showing the organization of SPB (Alp6-mCherry), kinetochores (GFP-CenpA), and microtubules (GFP-TubA). During mitosis, the SPB signals flank the mitotic spindle. The kinetochore (GFP-CenpA) signals cannot be distinguished from the initially strong signal of the spindle (GFP-TubA). However, as the mitotic spindle elongates and the signal is diffused toward the end of mitosis, the GFP-CenpA signals colocalize with those of Alp6-mCherry at the poles (see also Movie S4 at https://figshare.com/articles/MoCEN_movies/8282066). The images shown are maximum projections of 0.5-μm-spaced *z* stacks. Bars, 5 μm. Download FIG S2, TIF file, 2.3 MB. Copyright © 2019 Yadav et al. 2019 Yadav et al. This content is distributed under the terms of the Creative Commons Attribution 4.0 International license.

### Kinetochores undergo declustering-clustering dynamics during mitosis in M. oryzae.

**FIG 2:**
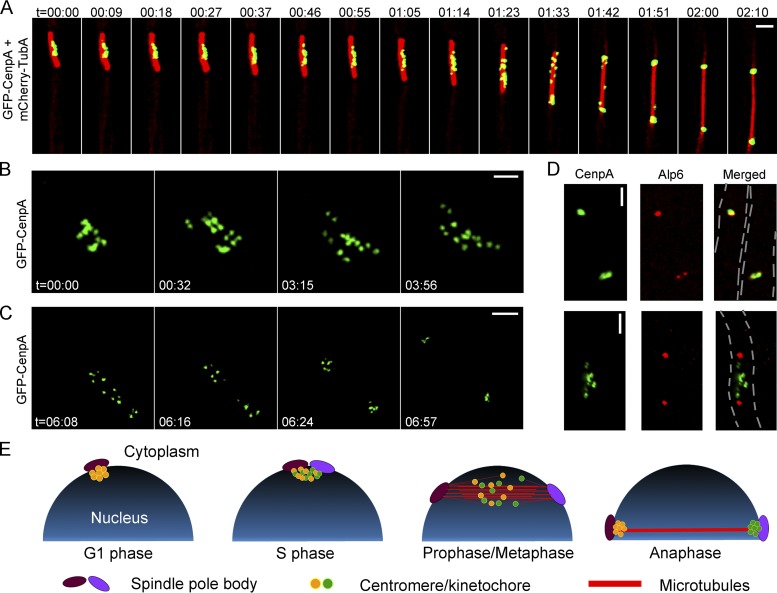
Kinetochores decluster momentarily but arrange randomly on the spindle axis before sister kinetochore separation during anaphase in M. oryzae. (A) Time-lapse imaging of strain MGYF07 cells exhibited that the GFP-CenpA signals separated from each other and moved along the mitotic spindle (mCherry-TubA) (see also Movie S1 at https://figshare.com/articles/MoCEN_movies/8282066). The images shown are maximum projections of 0.3-μm-spaced *Z* stacks. t = minutes:seconds. Bar, 2 μm. (B) High-resolution time-lapse images showing the declustering of kinetochores (GFP-CenpA) during the process of mitosis in strain MGYF01 (see also Movie S2 at https://figshare.com/articles/MoCEN_movies/8282066). The images were acquired with *Z* projections of 0.17-μm step size. t = minutes:seconds. Bar, 1 μm. (C) High-resolution time-lapse images of MGYF01 cells showing the segregation dynamics of sister kinetochores in daughter cells during the metaphase to anaphase transition and the final reclustering of kinetochores in postanaphase cells (see also Movie S3 at https://figshare.com/articles/MoCEN_movies/8282066). t = minutes:seconds. Bar, 2 μm. (D) Spatial organization of kinetochores (GFP-CenpA) and SPBs (Alp6-mCherry) in strain MGYF08 during the premitotic stage (upper panel) and early mitosis (lower panel). Bar, 2 μm. (E) A schematic depiction of centromere dynamics at specific stages of the cell cycle in M. oryzae. For simplification, chromosomes and astral microtubules are omitted in the schematic.

**FIG 3:**
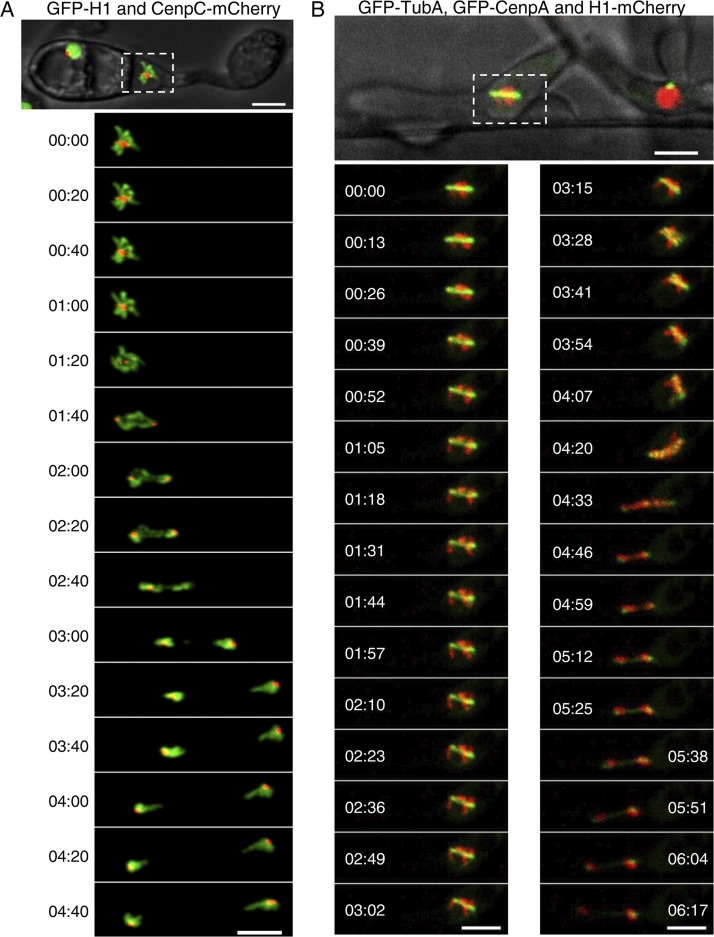
Subcellular localization and dynamics of CenpA and CenpC during pathogenic development in M. oryzae. (A) Time-lapse images showing a mitosis event during appressorium formation in CenpC-mCherry and GFP-histone H1-tagged strain B157 of M. oryzae. Conidia were incubated on the hydrophobic coverslip to allow appressorium development, and the mitotic division was recorded after 4 h postinfection (hpi), with images captured at 20-s intervals (see also Movie S5 at https://figshare.com/articles/MoCEN_movies/8282066). (B) Time-lapse images showing the localization of centromeres (GFP-CenpA), microtubules (GFP-TubA), and the nucleus (histone H1-mCherry) during mitosis in the invasive hyphae in M. oryzae. M. oryzae conidia were incubated on rice sheath, and the images were acquired at 44 hpi at 13-s intervals (see also Movie S6 at https://figshare.com/articles/MoCEN_movies/8282066). The epifluorescent confocal images shown here are maximum projections from Z-stacks consisting of 0.5-μm-spaced planes. Bars, 5 μm.

To study the cellular dynamics of kinetochores in M. oryzae, we localized microtubules by expressing an mCherry-TubA or GFP-TubA fusion protein and colocalized it with GFP-CenpA. During interphase, the microtubules are mostly localized throughout the cytoplasm (Fig. S2B). Live-cell imaging during mitosis revealed dispersed GFP-CenpA signals localized along the mitotic spindle (Fig. 2A; see also Movie S1 at https://figshare.com/articles/MoCEN_movies/8282066). Strikingly, the declustered dot-like signals of GFP-CenpA then segregated into two halves in a nonsynchronous manner. Once segregated, the signals began to cluster again and localized as two bright foci close to poles of the mitotic spindle. To further probe the dynamics of kinetochore segregation, we performed high-resolution imaging in mitotic cells expressing GFP-CenpA (Fig. 2B and C; see also Movies S2 and S3 at https://figshare.com/articles/MoCEN_movies/8282066). We observed that while the GFP-CenpA signals were spread out, they were localized in pairs, most likely representing the segregated kinetochore signals (Fig. 2B, time 00:32). We were able to count 14 discrete spots of GFP-CenpA corresponding to 14 kinetochores of the seven duplicated chromosomes. These results suggest that kinetochores remain largely unclustered in M. oryzae during mitosis. That idea was further supported by colocalization of GFP-CenpA with an SPB marker, Alp6-mCherry, during the mitotic stages (Fig. 2D). In premitotic cells, we observed two duplicated spots of Alp6-mCherry that colocalized with replicated clustered GFP-CenpA signals. During mitosis, GFP-CenpA signal localized as multiple puncta scattered between the two SPBs represented by Alp6-mCherry. After the division, the GFP-CenpA/kinetochores clustered again and localized adjacent to the SPBs (Fig. S2C; see also Movie S4 at https://figshare.com/articles/MoCEN_movies/8282066). Taking the data together, we conclude that kinetochores decluster during mitosis in M. oryzae and align along the mitotic spindle (Fig. 2E). Furthermore, we infer that an equatorial plate alignment of the kinetochores is not evident in M. oryzae, indicating a lack of a well-defined metaphase plate therein. Similar dynamics of the kinetochore and microtubules were observed in M. oryzae cells during pathogenic development and invasive growth *in planta* (Fig. 3; see also Movies S5 and S6 at https://figshare.com/articles/MoCEN_movies/8282066). On the basis of these observations, we propose a schematic model for kinetochore and SPB dynamics during the mitotic cycle in rice blast where the clustering/declustering cycle of kinetochores is likely dependent on their direct link to the SPBs (Fig. 2E). During mitosis, this link is likely broken and the clustering consequently perturbed. We infer that such a timely and dynamic cycle of kinetochore clustering/declustering is crucial for proper chromosome segregation in M. oryzae.

### Kinetochore protein binding identifies regional centromeres in M. oryzae.

**FIG 4:**
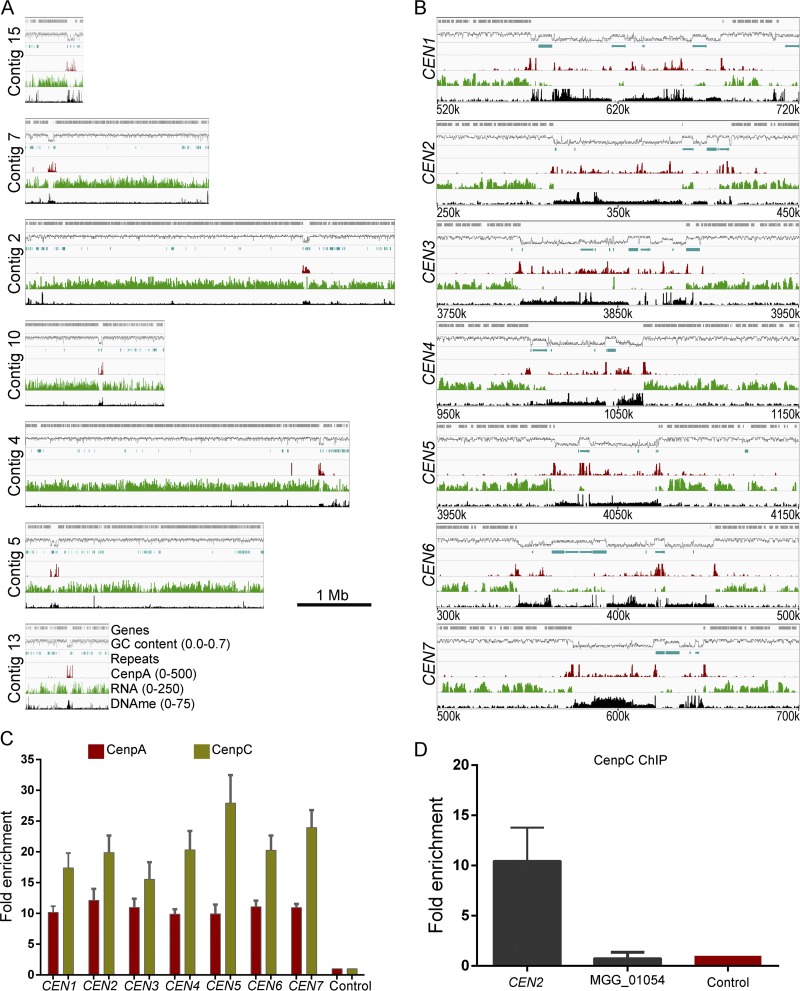
Identification of centromeres in M. oryzae. (A) Reads obtained from the GFP-CenpA ChIP-seq analysis in the cross-linked mycelia of strain MGYF01 identified one distinct enriched region on each of the seven contigs after alignment with the Guy11 genome assembly. CenpA-bound regions overlap AT-rich, poorly transcribed regions on each contig and harbor 5mC DNA methylation (see the text for details). The numbers in parentheses that appear after the indicated parameters represent the minimum and maximum values along the *y* axis. (B) Zoomed view of centromere regions in Guy11 depicting the presence of repeat elements, CenpA enrichment, poly(A) transcription, and DNA methylation (5mC) status in these regions. A 200-kb region spanning the centromere is shown for each chromosome. The only common defining sequence feature of centromeres is AT richness. (C) CenpA and CenpC ChIP-qPCR analysis of cross-linked mycelia of strains MGYF01 and MGYF02, respectively, confirmed the centromere identity of each of the seven chromosomes of Guy11. Each bar represents the extent of enrichment obtained by one primer pair amplifying a unique sequence of each CenpA-bound region identified from the ChIP-seq analysis, and fold enrichment values were normalized using a noncentromere region (ORF MGG_01917) as a control. Error bars represent standard deviations of results from three independent experiments. (D) ChIP-qPCR results showed that gene MGG_01045 is not enriched with CenpC as observed in CenpA ChIP-seq analysis.

CenpA binding is a hallmark of functional centromeres in eukaryotes (4, 20). We used GFP-CenpA as a tool for molecular identification of centromeres in the M. oryzae genome. We utilized chromatin immunoprecipitation (ChIP) assays followed by deep sequencing (ChIP-seq) of GFP-CenpA-associated chromatin fragments and aligned the reads on the recently published PacBio genome assembly of the wild-type Guy11 strain of M. oryzae (54). This analysis revealed seven distinct CenpA-rich regions across the genome, one each on seven different contigs (Fig. 4A and B) (Table 1; see also Fig. S3). The CenpA binding spans a 57-to-109-kb region, suggesting that M. oryzae possesses large regional centromeres. The centromere identity of these regions was further validated independently by binding of another evolutionarily conserved kinetochore protein, namely, CenpC. The independent ChIP-seq analysis performed using the fungal strain expressing CenpC-GFP confirmed the overlapping binding of CenpA and CenpC at each of these seven *CEN* regions (Fig. S4). We further validated the binding of both CenpA and CenpC to these regions using quantitative PCR (qPCR) in three independent experiments (Fig. 4C). We also noticed an extra albeit short region of 1,200 bp on contig 4 apart from the seven distinct peaks in CenpA ChIP-seq analysis. The enriched peak mapped to the gene encoding vacuolar morphogenesis protein AvaB (MGG_01045). Using specific ChIP-qPCR primers for this region, the aforementioned CenpA enrichment on contig 4 was deemed to be an artifact (Fig. 4D). Overall, the binding of two independent kinetochore proteins (CenpA and CenpC) at seven long regions confirmed that these are indeed authentic centromeres of the corresponding chromosomes in M. oryzae.

**TABLE 1:** Length and %GC content of centromeres in representative isolates of diverse host-adapted pathotypes belonging to *Magnaporthales*^*a*^

Centromere	Guy11		70-15^*b*^	FJ81278^*c*^		B71^*d*^		M. poae^*e*^	
	Contig and coordinates	%GC	Chromosome and coordinates	Contig(s) and coordinates	%GC	Chromosome or scaffold and coordinates	%GC	Scaffold accession no. and coordinates	%GC
*CEN1*	Contig 15: 571735– 678893 **(107,159)**	29.2	Chr 1: 4669580– 4690159 **(20,580)**	Contig 5: 942880– 1032698 **(89,819)**	26.8	Chr 1: 5247711– 5361150 **(113,440)**	29.4	GL876966: 1947276– 2058771 **(111,496)**	25.5
*CEN2*	Contig 7: 313767– 411084 **(97,318)**	30.8	Chr 2: 451419– 471909 **(20,491)**	Contig 1: 4838642– 4937646 **(99,005)**	30.5	Chr 2: 397914– 483923 **(86,010)**	27.4	GL876967: 2010105– 2095459 **(85,355)**	24.6
*CEN3*	Contig 2: 3795849– 3894639 **(98,791)**	33.0	Chr 3: 5534011– 5547746 **(13,736)**	Contig 13: 469353– 567903 **(98,551)**	34.6	Chr 3: 6398799– 6492490 **(93,692)**	25.8	GL876968: 291161– 385721 **(94,561)**	23.8
*CEN4*	Contig 10: 1000090– 1063263 **(63,174)**	30.5	Chr 4: 845436– 854002 **(8,567)**	Contig 14 [1– 38471] + Contig 3 [1–30023] **(68,694)**	30.0	Chr 4: 815420– 895650 **(80,231)**	24.8	GL876971: 2575155– 2652612 **(77,458)**	24.6
*CEN5*	Contig 4: 4014470– 4071774 **(57,305)**	28.0	Chr 5: 296487– 302043 **(5,557)**	Contig 4: 259241– 318616 **(59,376)**	28.8	Chr 5: 296404– 367652 **(71,249)**	24.8	GL876972: 961971– 1029283 **(67,313)**	23.3
*CEN6*	Contig 5: 343714– 452391 **(108,678)**	32.3	Chr 6: 3852307– 3861083 **(8,777)**	Contig 12: 708384– 794489 **(86,106)**	29.3	Chr 6: 5687241– 5758152 **(70,912)**	24.8	GL876975: 266207– 359379 **(93,173)**	24.2
*CEN7*	Contig 13: 573351– 645424 **(72,074)**	30.5	Chr 7: 2771164– 2774628 **(3,465)**			Chr 7: 3255069– 3336736 **(81,668)**	26.2	GL876978: 1–33204 **(33,204)**	37.1
						Scaffold 1: 40795– 109519 **(68,724)**	32.7	GL876979: 1–17025 **(17,025)**	24.6

a Centromere numbers are presented according to the isolate 70-15 genome assembly. Numbers in parentheses and highlighted in bold represent centromere lengths in base pair.

b The centromeres in isolate 70-15 contain breaks; hence, %GC content is not calculated for its centromeres.

c *CEN7* in FJ81278 could not be identified due to poor genome assembly.

d A predicted centromere for a minichromosome (in scaffold 1) of B71 is also listed.

e Two putative centromeres in M. poae are present at the end of two separate contigs. These two are listed as different centromeres here.

10.1128/mBio.01581-19.4 FIG S3 Identification of centromeres in wild-type M. oryzae strain Guy11. GFP-CenpA ChIP-seq revealed the identity of the centromeres in M. oryzae. Shown here is the CenpA binding pattern across contigs longer than 100 kb in Guy11 genome assembly. Contigs 1 to 17 (>500 kb in length) are shown at a different scale (bar = 1 Mb) compared to contigs 18 to 28 (<500 kb and >100 kb in length; bar = 100 kb) for visualization purposes. The presence of repeat elements, the GC content, and the levels of transcription and DNA methylation across all the contigs are also plotted. Download FIG S3, PDF file, 0.8 MB. Copyright © 2019 Yadav et al. 2019 Yadav et al. This content is distributed under the terms of the Creative Commons Attribution 4.0 International license.

10.1128/mBio.01581-19.5 FIG S4 CenpA and CenpC show overlapping binding at the centromere regions. Maps show binding of both CenpA and CenpC to the identified centromere regions. For both proteins, input (IP) graphs as well as subtracted-value graphs are presented. The binding of CenpC was found to be less enriched than that of CenpA, probably due either to experimental limitations or to its differential binding to centromeres. However, the binding regions of the two proteins significantly overlap. Download FIG S4, TIF file, 1.7 MB. Copyright © 2019 Yadav et al. 2019 Yadav et al. This content is distributed under the terms of the Creative Commons Attribution 4.0 International license.

**FIG 5:**
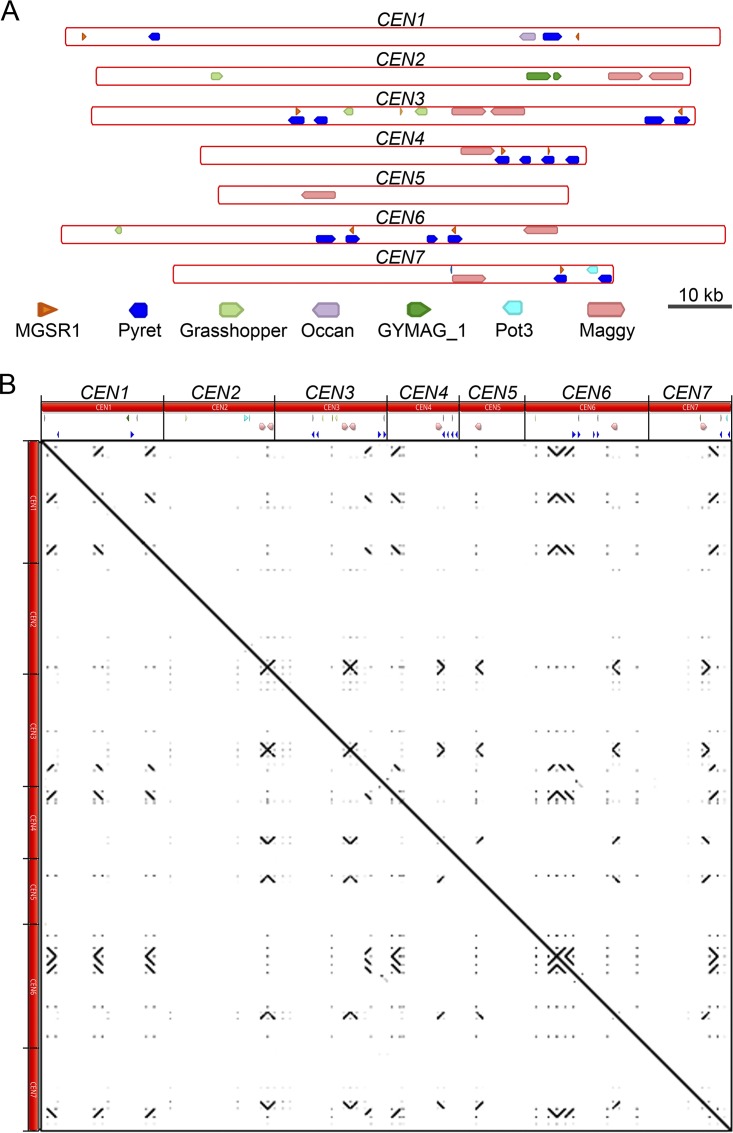
Centromere structure in M. oryzae (Guy11). (A) The well-characterized *Magnaporthe*-specific repeats were mapped to the centromere regions of the Guy11 genome to define the repeat content and organization of these regions. (B) Self-dot-plot analysis was performed for all the seven centromere regions, and the results are plotted. Each centromere sequence was compared with all centromeres, and the boundary of each centromere is marked to scale. Both the *x* axis and the *y* axis represent the lengths of all centromeres combined.

A detailed analysis revealed that the seven centromeres in M. oryzae comprise highly (≥67%) AT-rich sequences (Fig. 4B and Table 1). We did not find any other long AT-rich sequence in the rest of the Guy11 genome sequence (Fig. S3). Furthermore, such centromeres were found to be located in poorly transcribed regions that harbored 5mC DNA methylation in M. oryzae (Fig. 4A and B). The centromeres in M. oryzae were found to harbor a few repetitive elements (Fig. 4B) (see also Fig. 5A and Data Set S1 in the supplemental material). We analyzed the distribution of well-characterized *Magnaporthe*-specific repeat elements as well as that of generic repeat elements present in these regions (Data Set S1). However, none of these repetitive elements were either exclusive to the centromeres or common among the seven centromeres in M. oryzae. The most conserved element among centromeres was the Maggy retrotransposon, which was present in six of the seven centromeres. Sequence analysis of these regions did not reveal any evidence of interelement recombination even though some of these elements were found to be truncated (Data Set S1). Further in-depth analysis of these regions did not reveal any common DNA sequence motif or repeats as supported by the dot-plot analysis of all centromeres (Fig. 5B). We then examined the transcriptional status and base modifications associated with centromeric chromatin using the published RNA sequencing (RNA-seq) and bisulfite sequencing data (55, 56). On the basis of these results, we conclude that centromeres in M. oryzae do not share any common DNA sequence motif or repeat element(s) and that AT richness is likely the only defining sequence feature of all the centromeres in M. oryzae. We also infer that centromeres in M. oryzae are large and regional and lie within transcriptionally poor 5mC-rich DNA regions of the genome.

10.1128/mBio.01581-19.9 DATA SET S1 Analysis of repeats/repetitive elements in M. oryzae (Guy11) and M. poae genome assemblies. Download Data Set S1, XLSX file, 0.02 MB. Copyright © 2019 Yadav et al. 2019 Yadav et al. This content is distributed under the terms of the Creative Commons Attribution 4.0 International license.

### Centromere DNA sequences evolve rapidly in rice blast isolates.

**FIG 6:**
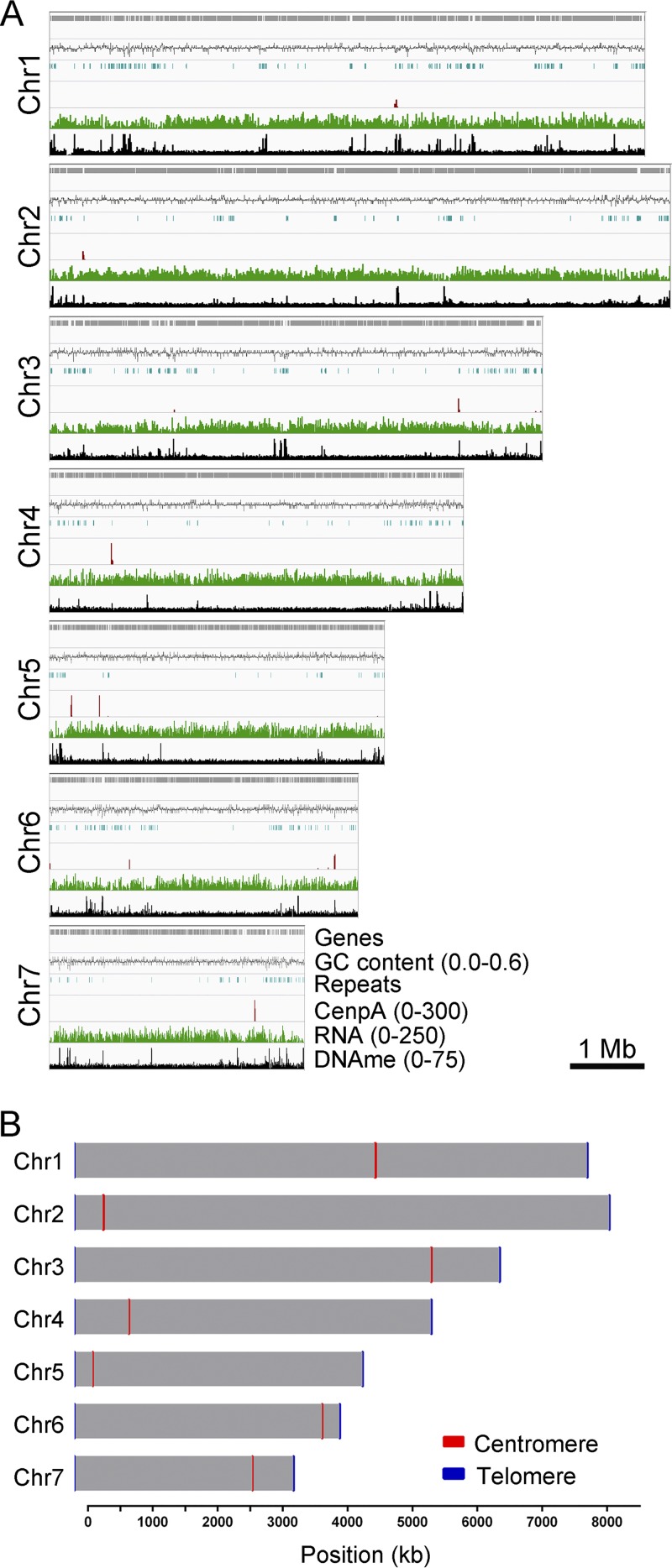
Identification of centromeres in M. oryzae strain 70-15 (MG8 assembly; Broad Institute). (A) Mapping of GFP-CenpA ChIP-seq reads to the reference MG8 genome assembly revealed the location of centromeres in reference strain 70-15 of M. oryzae. Repeats, RNA-seq reads, and bisulfite sequencing reads were also mapped and are represented here for the comparative analyses. (B) Map showing seven chromosomes of M. oryzae with centromere locations marked on each chromosome. The chromosome length along with centromere length obtained from the ChIP-seq analysis is plotted to the scale on the available chromosome-wide strain 70-15 genome assembly. However, telomeres are shown as 10-kb regions on either side for each chromosome for visualization purpose.

The MG8 genome assembly is based on the sequencing of M. oryzae isolate 70-15, which represents progeny of the Guy11 strain (49, 57, 58). The PacBio genome sequence of Guy11 provides nearly complete end-to-end chromosome-wide coverage of the 70-15 genome, the only chromosome-level sequence assembly (MG8) available for an M. oryzae rice pathogen (Fig. S5A). The breaks observed in the synteny map are due to contig-level genome assembly of Guy11 as well as sequence gaps present in the 70-15 assembly, despite claims of a chromosome-level assembly. Thus, we attempted to identify the centromere locations in the 70-15 genome by aligning the CenpA ChIP-seq reads onto the MG8 assembly. This analysis revealed seven distinct peaks, one on each chromosome (Fig. 6 and Table 1). We also observed two additional CenpA-enriched regions in unassembled supercontig 8.8 of MG8 assembly for 70-15 (Fig. S5B). Additionally, the identified centromere on chromosome 7 in this assembly matched the region previously predicted to harbor the centromere based on genetic analysis (59).

10.1128/mBio.01581-19.6 FIG S5 The M. oryzae strain 70-15 genome is syntenic with respect to the Guy11 genome. (A) Chromosome-wide synteny block map of the reference MG8 (strain 70-15) genome assembly with contigs from the Guy11 genome assembly. (B) Alignment of CenpA ChIP-seq reads to the unassembled region (supercontig 8.8) from the chromosome-wide MG8 genome assembly identified two significantly enriched regions. Download FIG S5, TIF file, 0.6 MB. Copyright © 2019 Yadav et al. 2019 Yadav et al. This content is distributed under the terms of the Creative Commons Attribution 4.0 International license.

**FIG 7:**
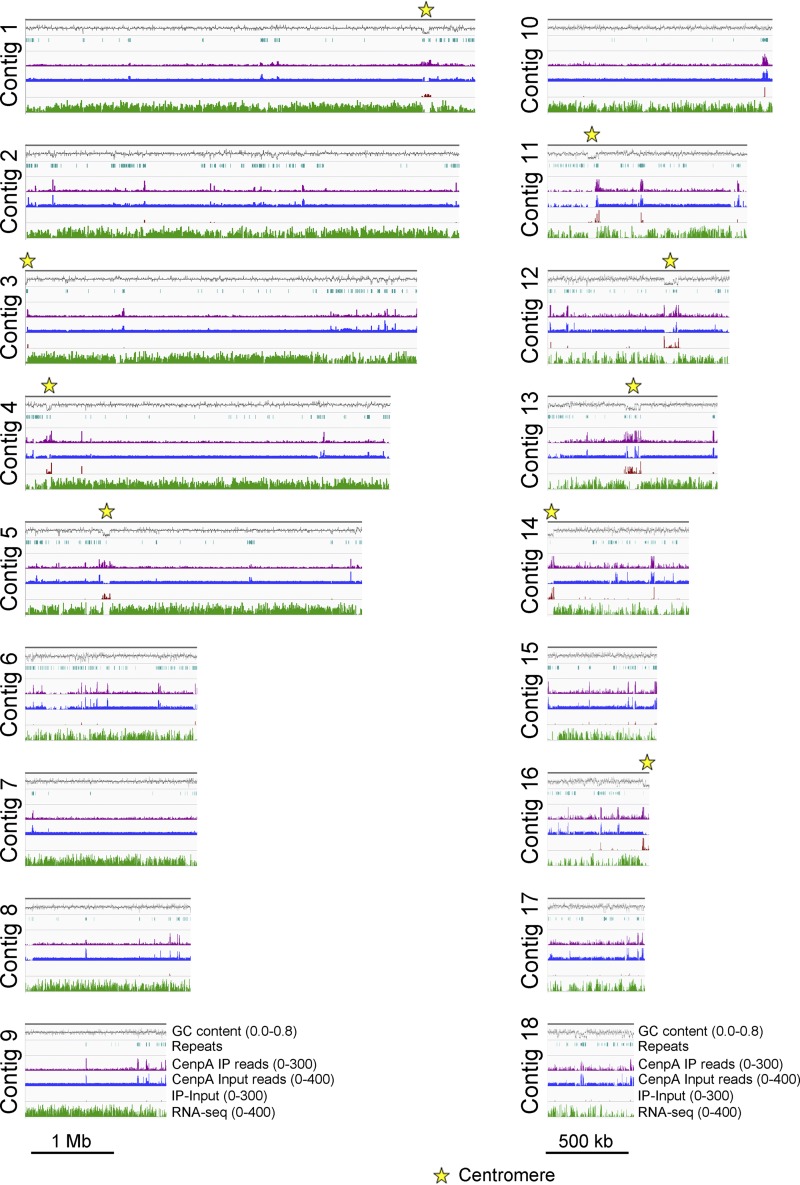
CenpA ChIP-seq read mapping identified centromere locations in M. oryzae isolate FJ81278. Graphs show the enrichment of CenpA in FJ81278 genome assembly. The enriched regions overlapped AT-rich regions. The locations of CenpA enriched centromeres are marked. The graphs are plotted with two different scales for the purpose of visualization.

Next, we analyzed the recently published PacBio genome assembly of the M. oryzae field isolate FJ81278 (54) to identify the centromere sequences and compare them with the 70-15 assembly. Mapping of CenpA ChIP-seq reads revealed nine distinct peaks in the FJ81278 genome assembly (Fig. 7) (Table 1). Three of these enriched regions were present at the end of three separate contigs (contigs 3, 14, and 16). By comparing genome assemblies of 70-15 and FJ81278, we concluded that contigs 3 and 14 are most likely parts of the same chromosome and that the CenpA-enriched regions observed in these two contigs represent a single centromere (*CEN4*). Synteny analysis also revealed that the CenpA peaks in contigs 11 and 16 belong to the same chromosome. However, contig 11 of FJ81278 assembly seems to be misassembled, since a part of this contig did not show synteny with any region of the 70-15 genome. Thus, we excluded this centromere (*CEN7*) region from further analysis.

**FIG 8:**
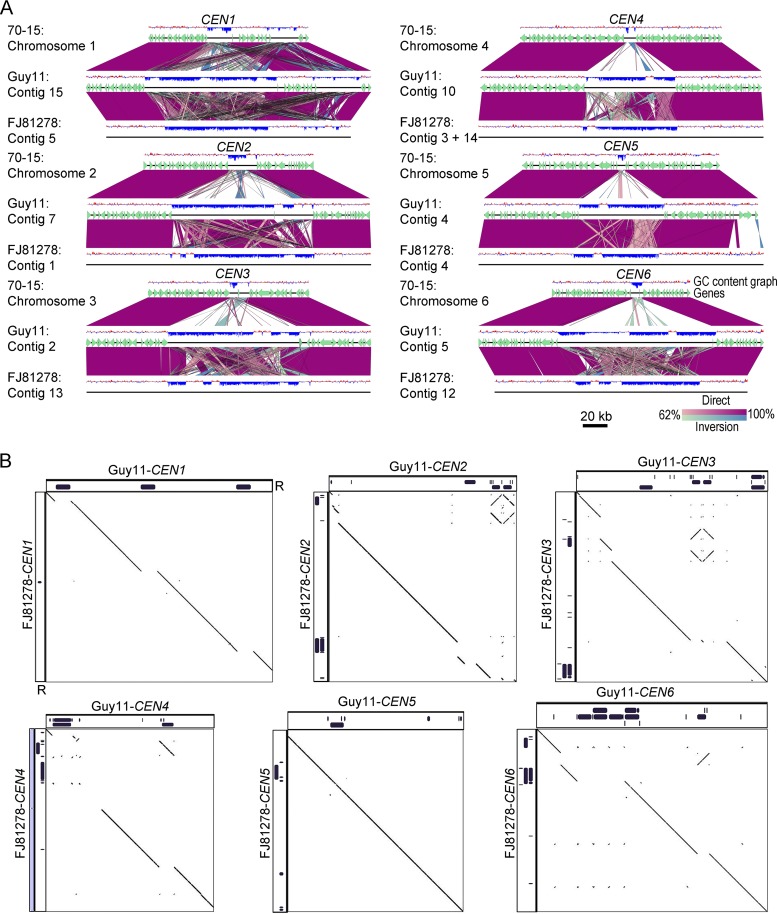
Centromere DNA sequences in M. oryzae isolates are similar but differ in repeat content. (A) Synteny analysis across centromeres and their flanking regions revealed the conservation of centromere flanking regions, indicating that the centromere location is maintained in different isolates of M. oryzae. The gene annotations for the FJ81278 assembly are not available and hence are not represented in the maps. This analysis also revealed that centromere sequences are largely excluded from the current MG8 genome assembly compared to that of Guy11 or FJ81278. A 200-kb region (corresponding to the Guy11 genome assembly) for each centromere is represented in the maps. A few centromere flanking genes were also found missing from Chr7 in the MG8 assembly. BLAST analysis revealed the presence of these genes in the unassembled supercontig 8.8 of the genome assembly. (B) Dot-plot analysis of respective centromeres revealed that centromere sequences share considerable similarities in Guy11 and FJ81278. As shown in the graphs, the breaks observed in the dot-plot analysis overlapped the presence/absence of repeat elements. The complete sequence of *CEN4* in FJ81278 was generated by fusing the two fragments, i.e., one each from contig 3 and 14. The individual fragments are shown using gray bars and are separated by a small thin black bar (equal to 100 bp). “R” denotes the repeat panels for both Guy11 and FJ81278.

We further compared the centromeres and flanking regions from the genome assemblies of Guy11, 70-15, and FJ81278. Detailed synteny analyses revealed that the centromere flanking regions are conserved among these three isolates, indicating that the overall position of centromeres is likely conserved in different strains/field isolates of M. oryzae (Fig. 8A). However, a major part of the centromere sequences is absent or misassembled in the 70-15 genome assembly compared to Guy11 and FJ81278. Note that the MG8 version of the 70-15 genome assembly is not complete and harbors a number of gaps. Thus, the rearrangements observed in comparisons of 70-15 to Guy11 are largely due to the aforementioned misassembly of the 70-15 centromere sequences. Additionally, we believe that some of the centromere sequences in the 70-15 genome assembly are part of unassembled supercontig 8.8 and contain the CenpA-enriched regions observed in this fragment (Fig. S5B). The centromere sequences of Guy11 and FJ81278 isolates shared a high level of conservation with certain rearrangements. To explore this further, we performed a pairwise comparison using sequences of the respective centromeres from the Guy11 and FJ81278 genomes. This analysis revealed that while most of the AT-rich sequence remained conserved between the two isolates, the repeat content varied significantly and accounted for almost all the observed rearrangements (Fig. 8B; see also Data Set S2). On the basis of these results, we infer that repetitive elements likely shape the structure of centromeres in different isolates of the rice blast fungus, even though such repeats may not be an integral part of centromeres *per se*.

10.1128/mBio.01581-19.10 DATA SET S2 Percent identity comparison of centromere sequences from Guy11, FJ81278, and B71. Download Data Set S2, XLSX file, 0.01 MB. Copyright © 2019 Yadav et al. 2019 Yadav et al. This content is distributed under the terms of the Creative Commons Attribution 4.0 International license.

### Intra- and interspecies comparison of *CEN* sequences in *Magnaporthales*.

**FIG 9:**
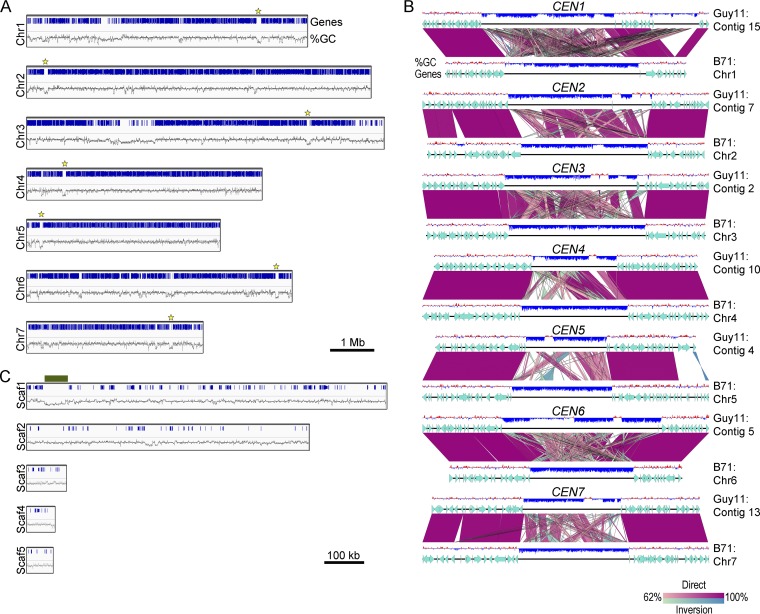
Predicted centromeres in the wheat blast B71 genome. (A) Chromosome maps showing the location of centromeres in the genome of the B71 isolate of wheat blast. The AT-rich, gene-free centromere regions are marked by a yellow star in each chromosome. (B) Synteny analysis of centromeres and their flanking regions between the Guy11 and B71 genomes showed conservation of centromere locations therein. A 200-kb region is shown with respect to the Guy11 genome assembly. (C) Scaffolds (Scaf1 to Scaf5) representing the minichromosome in B71 were analyzed and are depicted with the corresponding genes and AT richness graphs. A long AT-rich region (marked with a dark green bar) in scaffold 1 (position 40795 to position 109519) was identified and represents the putative centromere in the minichromosome in wheat blast.

The results of analysis of different isolates of M. oryzae further validated that centromeres in this species are comprised of long AT-rich and transcription-poor regions. Using these parameters, we decided to predict the centromeres in wheat blast isolate B71 (Triticum pathotype of M. oryzae*;* MoT) as well as in M. poae, a root-infecting pathogen that belongs to the *Magnaporthaceae* family (47). The genome of wheat blast isolate B71 was assembled to the chromosome level and was found to exhibit a few chromosomal rearrangements compared to the rice blast 70-15 genome assembly (60). We identified seven putative centromeres, one in each chromosome, in the B71 genome. These centromeres in the wheat blast genome were long AT-rich regions (Fig. 9A and Table 1). We then analyzed the centromere flanking regions between two genomes and found that the centromere locations are conserved between the rice blast and wheat blast strains (Fig. 9B). Further analysis revealed that the centromeres in wheat blast B71 also harbor a few repeats but are defined primarily by AT richness, similarly to those observed in the rice blast isolates. We analyzed scaffold 1 representing the minichromosome in B71 (60) and identified a putative centromere based on a long AT-rich region (bp 40795 to 109519) (Fig. 9C and Table 1).

**FIG 10:**
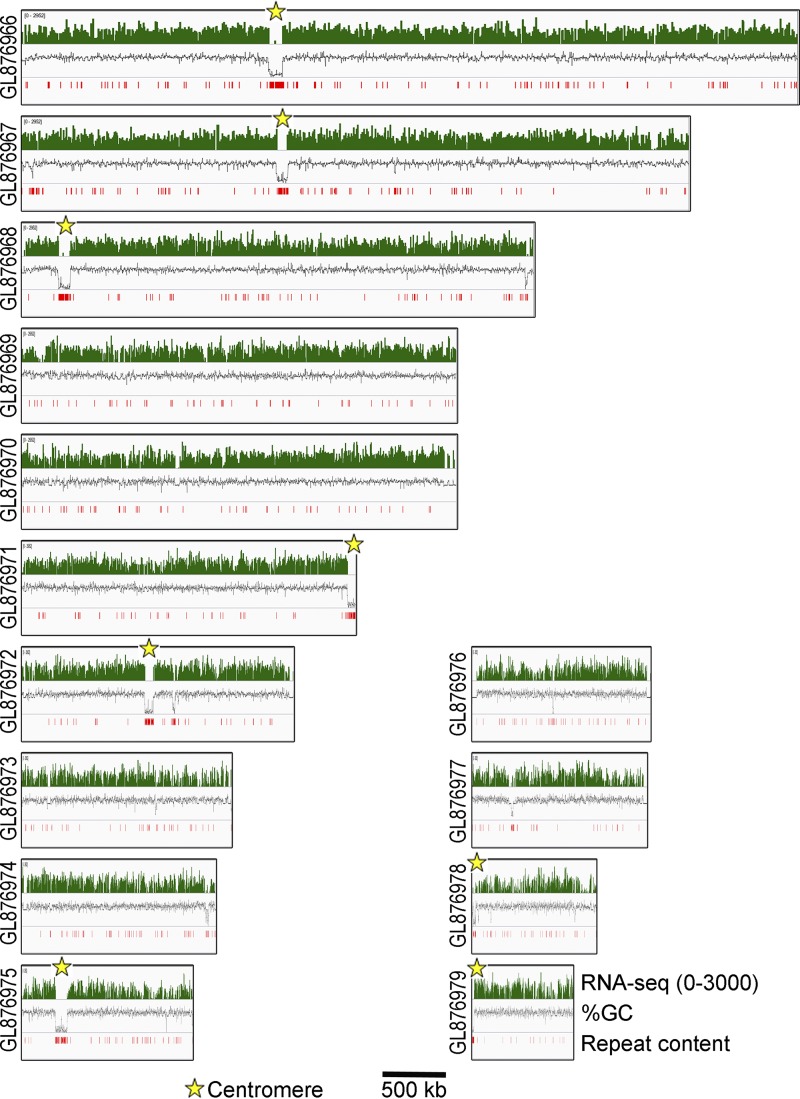
*In silico* centromere identification in Magnaporthiopsis poae. On the basis of the RNA-seq read, AT richness, and repeat content data, centromeres were identified (as marked) in the M. poae genome, and the graphs showing the same are plotted for the M. poae genome assembly. Only contigs longer than 500 kb are represented here.

Next, we extended our *in silico* analysis to M. poae, a distinct species within the *Magnaporthales* (43), and identified eight putative centromere regions across its genome (Fig. 10) (Table 1). Three of the eight putative *CEN* regions are present at the end of different contigs. Since the chromosome number in M. poae is not established, it is uncertain whether all of these AT-rich regions represent *bona fide* centromeres in M. poae. We also found that these putative centromeres in M. poae harbor more repetitive DNA sequences than those in M. oryzae even though the genomic repeat content of M. poae is only 1.1% compared to 10.1% in M. oryzae (Data Set S1). Unlike the different isolates of M. oryzae that share a high level of centromere sequence conservation, the centromere sequences in M. oryzae and M. poae are highly divergent. On the basis of these results, we conclude that centromere DNA sequences in the members of *Magnaporthales* are rapidly evolving, whereas the properties of centromeric chromatin are likely conserved between the two species.

## DISCUSSION

The effects of BLAST diseases caused by M. oryzae are exceedingly disastrous not only with respect to rice production worldwide but also with respect to wheat and other graminaceous crops (45, 48). Despite being such an important plant pathogen, the fundamental cellular process of chromosome segregation is not well understood in this organism. In this work, we attempted to study the chromosome segregation machinery in M. oryzae at the molecular level. We tagged two evolutionarily conserved key kinetochore proteins in M. oryzae and studied their dynamics during different phases of the cell cycle at various developmental stages. We further identified the genomic loci that act as centromeres in this filamentous fungus. On the basis of the results of comparisons of centromeric sequences among different host-adapted lineages of M. oryzae and in the related species (M. poae), centromeres appear to be rapidly evolving in the *Magnaporthe* species complex, similarly to those reported in several fungal genera (6, 8, 10, 17, 61, 62).

Kinetochores cluster together in a single locus at the nuclear periphery in many fungi. This locus is often referred to as the CENP-A-rich zone or CENP-A cloud (14, 63). It has been proposed that such a nuclear subdomain with a high concentration of CENP-A favors centromere seeding on the chromosomal regions in close proximity to it, in the absence of a centromere-specific DNA sequence. In most budding yeasts, kinetochores are clustered throughout the cell cycle; the exception is C. neoformans, which shows clustered kinetochores only during mitosis (28). The kinetochore dynamics in M. oryzae is found to be similar to that in the “fission” yeast rather than that of the budding yeast species. It is possible that mitotic declustering of kinetochores is a feature of all yeasts/fungi that divide by septum formation. However, a more detailed analysis of kinetochore behavior in filamentous fungi such as N. crassa and Z. tritici will be useful to establish this link. It is noteworthy that Z. tritici does not have a single centromere cluster and that the kinetochores are arranged in multiple chromocenters, a process observed in some plant species (35, 64). We also observed that kinetochores align along the mitotic spindle in M. oryzae, though proper metaphase plate formation was not evident. A similar kinetochore arrangement was also observed in a basidiomycete, C. neoformans (28). The presence of a metaphase plate is a hallmark feature of mitosis in both animals and plants, but is difficult to determine or undetected in fungi. However, the presence of mitotic chromosome alignment in two evolutionarily distant fungal species suggests the existence of a transient metaphase-plate like structure (28), an arrangement alternative to the metaphase plate, across the fungal kingdom. In addition, colocalization of kinetochore proteins and SPBs revealed a close association between the two as observed in S. pombe (34). Our results also suggest that a direct interaction between the SPBs and kinetochores may facilitate kinetochore clustering. The SPB-kinetochore interaction has been explored in other fungi, and the results led to the identification of several uncharacterized proteins (31, 37, 65–67). It remains to be seen whether or not such interactions occur in M. oryzae as well.

Centromere DNA sequences, despite being associated with a conserved and essential function, are highly divergent across species (3). The centromeres identified in M. oryzae further add to this diversity of centromere sequences. Our results show that centromeres in M. oryzae, similarly to those reported in N. crassa, are long and AT rich; however, the centromeres are shorter in M. oryzae (57 to 109 kb) than in N. crassa (150 to 300 kb) (17, 18). The DNA methylation pattern observed in M. oryzae is similar to that in N. crassa, as it is present at multiple loci in both the organisms and thus differs from that of C. neoformans, where DNA methylation is restricted to centromeres and telomeres only (8, 18). Additionally, a specific pattern of centromeric histone binding has been reported in N. crassa, but no such pattern exists in M. oryzae. Since centromere DNA sequences are generally rich in repeats, they are poorly assembled, which restricts finer analysis of *CEN* DNA sequence. For example, centromeres in Fusarium graminearum are proposed to be AT rich, similarly to those of M. oryzae and N. crassa (17). However, the exact nature of the centromere sequence of these regions in F. graminearum remains unknown due to sequence gaps in the genome assembly. Likewise, the major parts of the predicted centromere sequences are either misplaced or absent in the currently available 70-15 genome assembly of M. oryzae. In contrast, the centromere regions in the Guy11 genome assembly are intact and have been completely sequenced using PacBio long-sequencing reads. We verified the full coverage of such centromere regions with the original PacBio raw reads in both Guy11 and FJ81278 (Fig. S6), thus confirming the integrity of the centromere sequences in these genomes.

10.1128/mBio.01581-19.7 FIG S6 Most centromere sequences in M. oryzae do not contain sequence gaps. (A) A read alignment profile showing uniform coverage of centromeres and flanking regions in the Guy11 PacBio genome assembly. Original PacBio reads were mapped to the genome assembly to determine the coverage across these regions. (B) A read alignment profile for the centromeric regions of FJ81278 genome is shown. *CEN4* and *CEN7* are broken in the current genome assembly and hence were omitted from this analysis. A 200-kb region is shown in the maps for each of Guy11 and FJ81278. Download FIG S6, TIF file, 2.1 MB. Copyright © 2019 Yadav et al. 2019 Yadav et al. This content is distributed under the terms of the Creative Commons Attribution 4.0 International license.

The common feature of M. oryzae centromeres is AT richness, which is also observed in some centromeres of other filamentous fungi such as N. crassa and Z. tritici (17, 18, 35). The centromeres in S. pombe, C. neoformans, and C. albicans are not AT rich (8, 11, 62) and hence differ from the centromeres in M. oryzae. Apart from filamentous fungi, AT-rich centromeres are present in other fungal/yeast species such as Malassezia sympodialis, albeit these regions are significantly smaller than those of M. oryzae (42). Centromere DNA element II (CDEII) of point centromeres present in the budding yeast S. cerevisiae is also highly AT rich (68). A recent study reported the presence of AT-rich centromeres of various lengths in diatoms (40). Furthermore, the 171-bp alpha satellite repeat DNA present in human centromeres is also AT rich in nature (69). Overall, these results suggest that AT richness favors centromere function in many organisms. Intriguingly, *in vitro* experiments suggest that CENP-A binds with lower affinity to an AT-rich DNA sequence (70). In contrast, the same study also revealed that the CENP-A chaperone Scm3 has higher affinity for AT-rich sequences. With more AT-rich centromeres being characterized, identifying the exact role of AT-rich sequences in centromere function is critical.

Regional centromeres of many organisms, including M. oryzae, do not share any common DNA sequence motifs. Rather, non-DNA sequence determinants mark centromeres in an epigenetic manner in many organisms. Some epigenetic determinants of centromere identity in fungi include early replicating regions of the genome (71–73), proximity to DNA replication origins (15), DNA replication initiator proteins (74), homologous recombination-repair proteins (15, 75), and proteins that facilitate kinetochore clustering by tethering kinetochores to SPBs (31, 65). Factors that favor local folding and looping of chromatin may also add to the process of centromere specification (4, 14, 76). Many histone posttranslational modifications as well as DNA methylation have been known to be associated with centromeres. For example, H3K4diMe has been identified as a mark for centromere chromatin in *Drosophila* (77). Similarly, H3K9diMe is specifically associated with centromeres in S. pombe and C. neoformans (8, 39). Future studies will provide information on whether a similar correlation is present in M. oryzae as well. DNA methylation data are publicly available for M. oryzae, analysis of which did not reveal centromere-specific enrichment for this epigenetic modification. Understanding the role of these as well as other posttranslational modifications will be required to further establish the importance of epigenetic factors in centromere identity and/or function in M. oryzae. Repeats and transposons have been shown to play an essential role in centromere evolution (78–80). Previous reports in M. oryzae suggested the presence of multiple clusters of repeat elements across the genome (55, 59). Those studies also proposed that repeats play an important role in M. oryzae genome evolution and its association with the host. In this study, we found that the centromere location is close to these repeat clusters in some but not all chromosomes. Our results raise the possibility that centromere sequences in M. oryzae are prone to repeat-mediated evolution.

A comparison of two M. oryzae isolates, Guy11 and FJ81278, revealed that while the overall *CEN* DNA sequences of the two rice blast isolates are very similar, the repeat content at the centromeres of orthologous chromosomes differed greatly. It is known that the centromere DNA sequences can be different among isolates of N. crassa (17). Analyses based on the *CEN* sequences identified here would pave the way for a more detailed comparative analysis of centromeres in diverse isolates of M. oryzae. Such analyses will provide valuable insights into centromere evolution in this species and the potential impact of host factors on this process. A genome analysis comparing M. oryzae and M. poae revealed a higher density of repeats in the predicted *CEN* regions in the latter. Overall, these results suggest that while the centromere DNA sequence properties, rather than the DNA sequence *per se*, remain conserved in members of the fungal order *Magnaporthales*, the centromere architecture is divergent and might have been shaped by the repeat elements. Further studies will provide more insights into the evolution of centromere DNA sequences and its possible link to host adaptation and variability in virulence within the members of this important family of cereal killers.

## MATERIALS AND METHODS

### Fungal strains and culture conditions.

Wild-type M. oryzae strain Guy11 (MAT1-2; a kind gift from the M. H. Lebrun group, France) was used as the parent strain for all the experiments conducted in this study (except for the results shown in Fig. 3 and in Movies S5 and S6 at https://figshare.com/articles/MoCEN_movies/8282066 that were obtained in experiments performed using the B157 strain). The fungal strains were propagated on prune agar (PA) medium or complete medium (CM) as described previously (81). For conidiation, fungal strains were grown on PA plates at 28°C for 2 days in the dark followed by exposure to continuous light at room temperature for 5 days. Conidia were harvested using an inoculation loop by gently scraping the culture surface in sterile distilled water. The resulting conidial suspension was filtered through two layers of Miracloth (Calbiochem) to remove mycelial debris and was adjusted to obtain the required concentration after counting was performed with a hemocytometer. Agrobacterium tumefaciens-mediated transformation (ATMT) of M. oryzae was carried out as described previously (81, 82). Transformants were screened for antibiotic resistance using the respective selection media, i.e., CM with 250 μg/ml hygromycin B, basal media with 50 μg/ml ammonium glufosinate (Basta) or chlorimuron-ethyl (sulfonylurea) for selection. Transformants were verified for correct genomic integration by diagnostic PCR and sequencing. The strains thus validated and used in this study are listed in Table S1A in the supplemental material. The plasmids and primers used for epifluorescence labeling in M. oryzae strains are listed in Table S1B and C, respectively. Detailed information regarding construction of the plasmids is available in Text S1 in the supplemental material.

10.1128/mBio.01581-19.1 TEXT S1 Supplemental methods. Download Text S1, DOCX file, 0.01 MB. Copyright © 2019 Yadav et al. 2019 Yadav et al. This content is distributed under the terms of the Creative Commons Attribution 4.0 International license.

10.1128/mBio.01581-19.8 TABLE S1 List of fungal strains, plasmids, and primers used in this study. Download Table S1, DOCX file, 0.02 MB. Copyright © 2019 Yadav et al. 2019 Yadav et al. This content is distributed under the terms of the Creative Commons Attribution 4.0 International license.

### Microscopy and image processing.

Unless otherwise stated, live-cell microscopy imaging was performed on a motorized inverted Nikon Eclipse Ti-E microscope with a Perfect Focus system equipped with a Yokogawa CUS-X1 spinning-disk confocal system and a CFI Plan Apo VC 100×/1.4 numerical aperture (NA) oil lens objective. The images were captured using a 16-bit digital Orca-Flash4.0 scientific complementary metal oxide semiconductor (sCMOS) camera (Hamamatsu Photonics KK) and laser illumination at 491 nm and 100 mW (for green fluorescence) and at 561 nm and 50 mW (for red fluorescence) operated by MetaMorph Premier software (Ver. 7.7.5; Universal Imaging). The maximum-projection images were obtained from Z stacks of 0.5-μm-spaced sections using the built-in MetaMorph module. Image processing was performed using Imaris (Bitplane) and Fiji (https://imagej.net/Fiji). A Live-SR module (Gataca Systems), which is based on an optically demodulated structured illumination technique with online processing, was additionally mounted on the same spinning-disk confocal system during acquisition of the images that appear in Fig. 2A. The high-resolution images that appear in Fig. 2B and C were acquired using an Andor Dragonfly high-speed confocal microscope equipped with an iXon888 electron microscopy charge-coupled-device (EMCCD) camera and a 100× oil lens objective. The raw images were immediately processed using the integrated Fusion software and the in-built deconvolution feature.

### Chromatin immunoprecipitation.

The ChIP experiment was performed using a previously described protocol (83) with a few modifications. An M. oryzae strain expressing GFP-CenpA or CenpC-GFP fusion protein was grown in 150 ml complete media for 3 days at 28°C with continuous shaking at 150 rpm. Fungal mycelia were collected using two layers of Miracloth (Calbiochem), and the harvested mycelia were washed with sterile water. Mycelia were cross-linked by suspending them in 1% formaldehyde solution–20 mM HEPES (pH 7.4) for 20 min with continuous shaking at 100 rpm. Glycine was added to the suspension at a final concentration of 0.125 M, and the mix was further incubated at room temperature for an additional 10 min. Cross-linked mycelia were harvested using Miracloth and rinsed with water. The excess water was removed by gently patting the mycelium mass with paper towels followed by snap-freezing in liquid nitrogen. The frozen mass was then stored at −80°C until use. For each ChIP experiment, 80 to 100 mg of frozen mycelia was ground in liquid nitrogen using a mortar and pestle, and powdered mycelia were resuspended in 1 ml of nucleus isolation buffer (10 mM MES-KOH [morpholineethanesulfonic acid-KOH], 10 mM NaCl, 10 mM KCl, 2.5 mM EDTA [pH 8.0], 250 mM sucrose, 0.1 mM spermine, 0.5 mM spermidine-free base, 1 mM dithiothreitol [DTT]). Nuclei were separated from debris by filtering them through two layers of Miracloth and were pelleted by centrifugation at 13,000 rpm for 10 min at 4°C. The pellet was resuspended in 1 ml of lysis buffer (50 mM HEPES [pH 7.5], 150 mM NaCl, 1 mM EDTA, 0.1% Na-deoxycholate, 1% Triton X, 0.1% SDS). The resuspended nuclei were subjected to sonication using a Bioruptor (Diagenode) for 60 cycles of bursts of 30 s on and 30 s off at the high setting, and the fragmented chromatin (300 to 600 bp) was isolated by centrifugation at 13,000 rpm and 10 min at 4°C. A part of the chromatin fraction (100 μl) was used for input DNA (I) preparation, and the remaining chromatin solution was divided into two halves (450 μl each). A 20-μl volume of GFP-TRAP beads (ChromoTek) was added for immunoprecipitation in one of the tubes (+), while the other tube was incubated with 20 μl of blocked agarose beads (ChromoTek) for use as the negative control (−). The tubes were incubated at 4°C for 12 h on a rotator. The beads were then washed, and bound chromatin was eluted in 500 μl of elution buffer (1% SDS, 0.1 M NaHCO_3_). All three fractions (the I, +, and − fractions) were de-cross-linked, and DNA was isolated using phenol-chloroform extraction followed by ethanol precipitation. The precipitated DNA was air dried and dissolved in 25 μl of MilliQ water containing 25 μg/ml RNase (Sigma-Aldrich). I and + samples were subjected to ChIP-seq analysis for GFP-CenpA ChIP in M. oryzae. Both the GFP-CenpA and CenpC-GFP ChIP samples (I, +, and −) were subjected to qPCR with centromere-specific primers along with a noncentromeric primer set. Three independent ChIP experiments, followed by three PCR replicates for each, were performed for both CenpA and CenpC to calculate the fold enrichment of these proteins at the centromeres. The calculated fold enrichment at the centromere was plotted using GraphPad Prism software.

### Analysis of sequencing data.

GFP-CenpA and CenpC-GFP ChIP sequencing was performed at Clevergene Biocorp. Pvt. Ltd., Bengaluru, India. More than 20 million 150-bp paired-end reads were obtained for each sample. The reads were mapped to the Guy11 PacBio genome (GCA_002368485.1), the 70-15 genome (GCA_000002495.2) and the FJ81278 genome assembly (GCA_002368475.1) using Geneious 9.0 under default conditions. Each read was allowed to map randomly only once anywhere in the genome. The alignments were exported to bam files and were sorted and visualized using Integrative Genomics Viewer (IGV, Broad Institute). The images from IGV were imported into Adobe Photoshop (Adobe systems) and scaled for the purpose of representation.

RNA-sequencing data (SRR1568068) from a previous study were downloaded from the NCBI website and aligned to genomes using RNA-seq alignment default parameters in Geneious 9.0. Similarly, bisulfite sequencing data (SRR653493) were obtained from NCBI, and the reads were aligned to genomes using the Geneious default aligner. The aligned files were exported into bam files and visualized using IGV. The RNA-seq reads were plotted in logarithmic scale for the purpose of visualization. GC content was calculated using Geneious 9.0 with a sliding window size of 200 bp. The data were exported as wig files and further visualized using IGV.

The GC content and RNA-seq data were plotted for M. poae as described for M. oryzae. The RNA-seq data were downloaded from NCBI (SRR057701) and were aligned to the reference genome (GCA_000193285.1).

The annotations of the repeats presented in this study were done based on repeat sequences described in a previous study (47). The BLASTn analysis was carried out using these repeat sequences, the results were sorted, and hits with 100% query coverage were extracted. The hits were mapped onto the respective genome assemblies of Guy11, FJ81278, B71, and M. poae and visualized using IGV.

To check the sequence integrity of the centromere regions, we mapped the original PacBio reads from Guy11 and FJ81278 to their respective genome assemblies. The fastq sequence files were aligned to genome files using Burrows-Wheeler Aligner (BWA) to obtain bam alignment files. The bam alignment files were then visualized using Geneious 9.0 to observe the read coverage of centromere regions. A 200 kb-window that included the centromere was selected and is shown in Fig. S6.

### Synteny analysis.

The synteny analysis of comparisons between centromere flanking regions was performed using Easyfig software (84) (http://mjsull.github.io/Easyfig/). The graphs were plotted under default conditions (except with respect to the color settings), and GC percentage was plotted as 200-bp sliding window. The dot-plot analysis of centromere sequences was carried out using gepard software with a 100-bp window (85).

### Data availability.

The ChIP-seq reads have been deposited under NCBI BioProject accession identifier (ID) PRJNA504461.
