# Supplementary figures and images for: Cellular Dynamics and Genomic Identity of Centromeres in Cereal Blast Fungus
Source: mBio. 2019 Jul 30;10(4):e01581-19. doi: 10.1128/mBio.01581-19 (PMC6667624; doi:10.1128/mBio.01581-19)

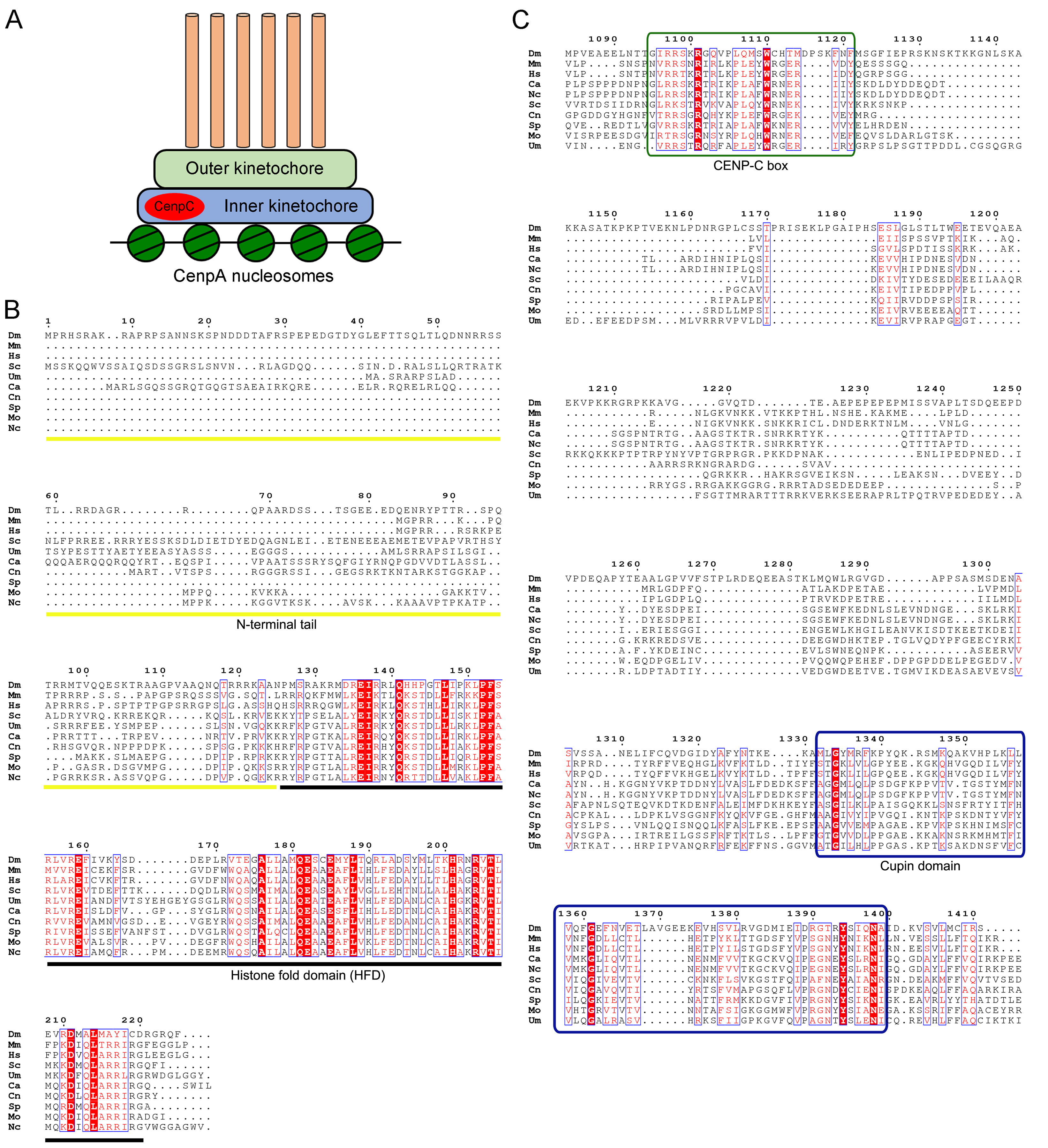

Supplement: FIG S1 [file mBio.01581-19-sf001.tif]

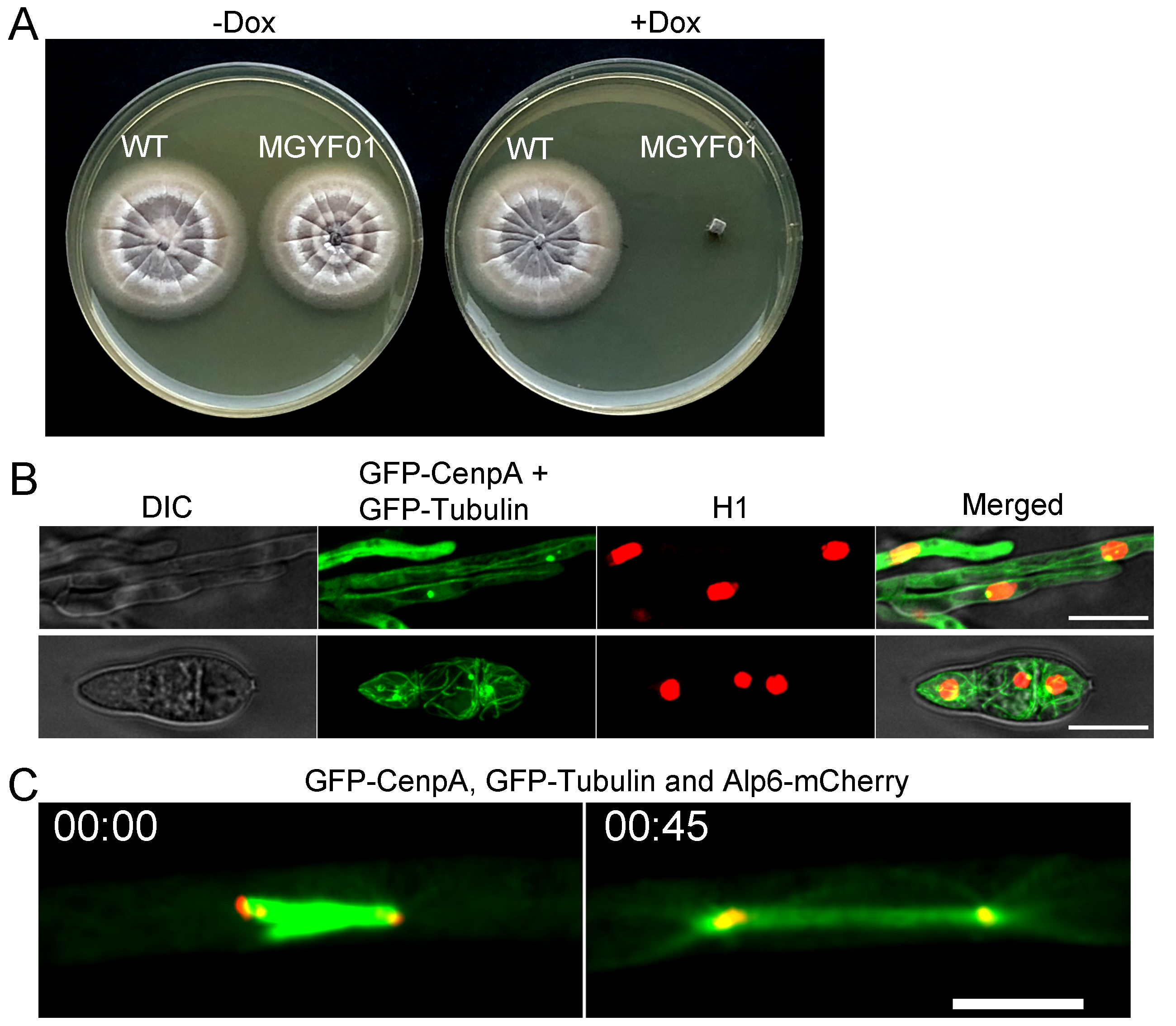

Supplement: FIG S2 [file mBio.01581-19-sf002.tif]

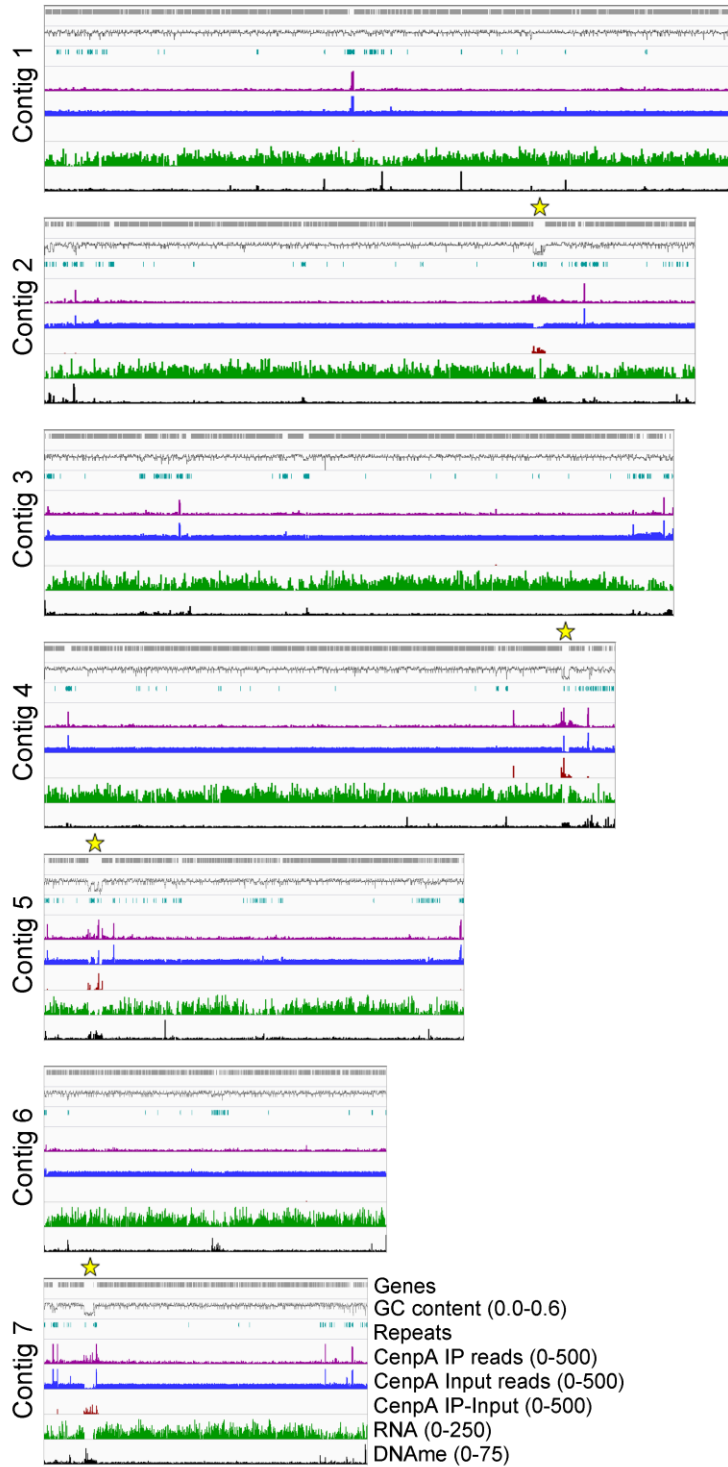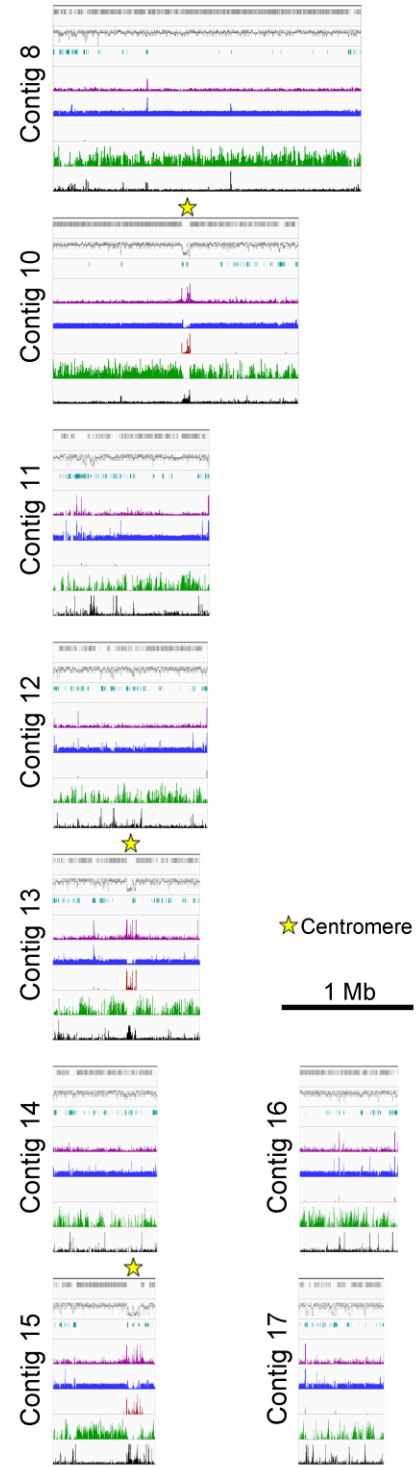

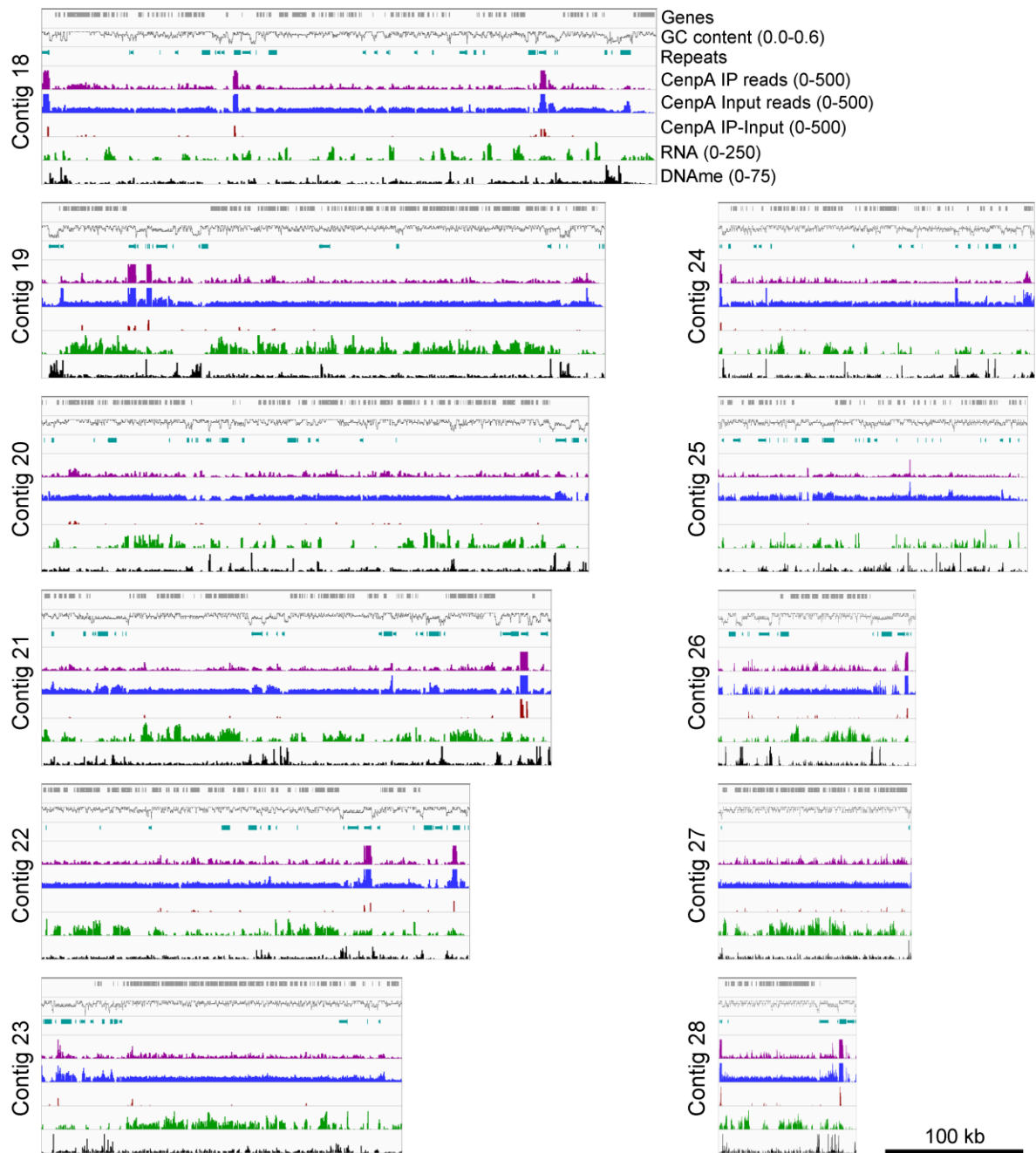

Supplement: FIG S3 [file mBio.01581-19-sf003.pdf]

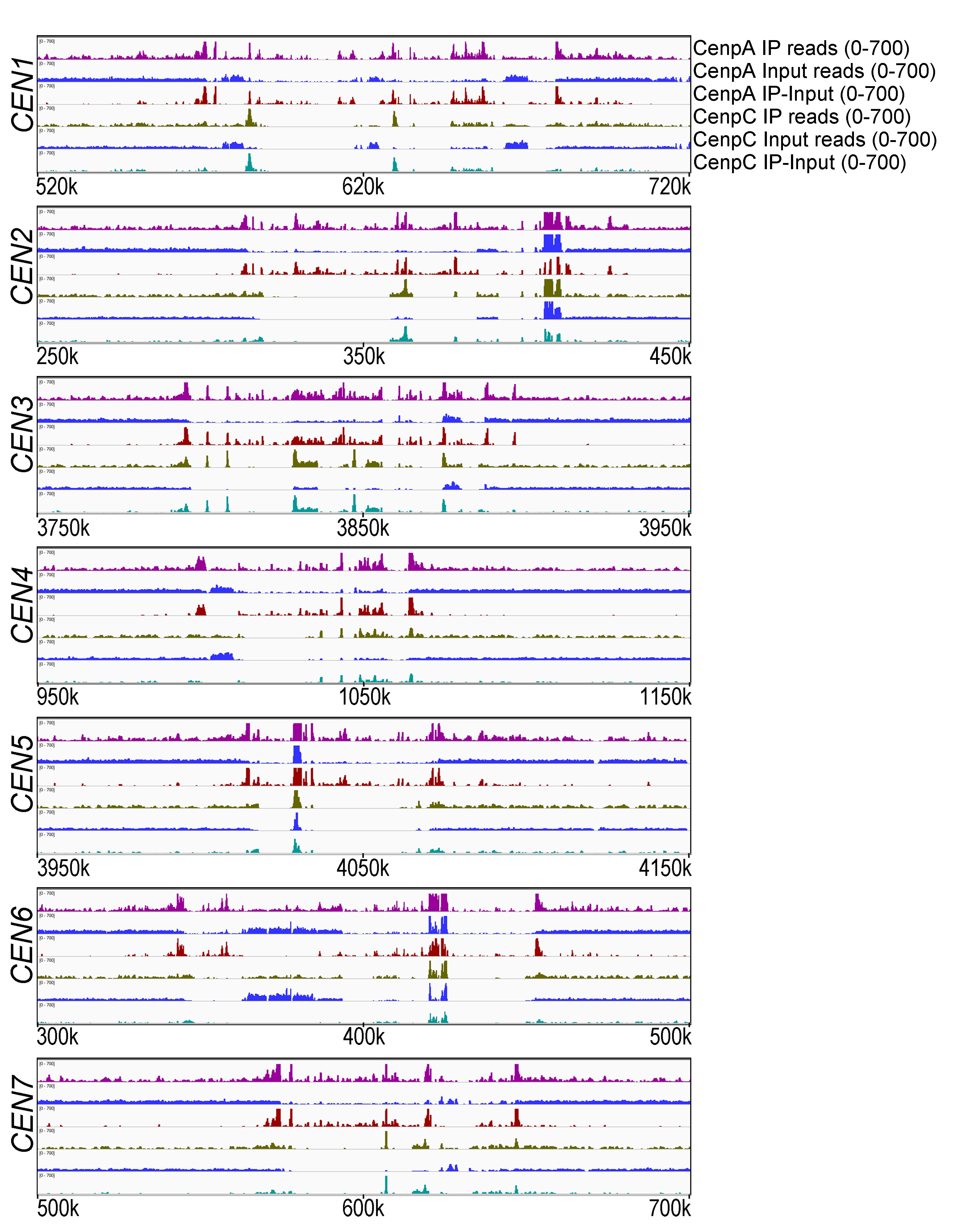

Supplement: FIG S4 [file mBio.01581-19-sf004.tif]

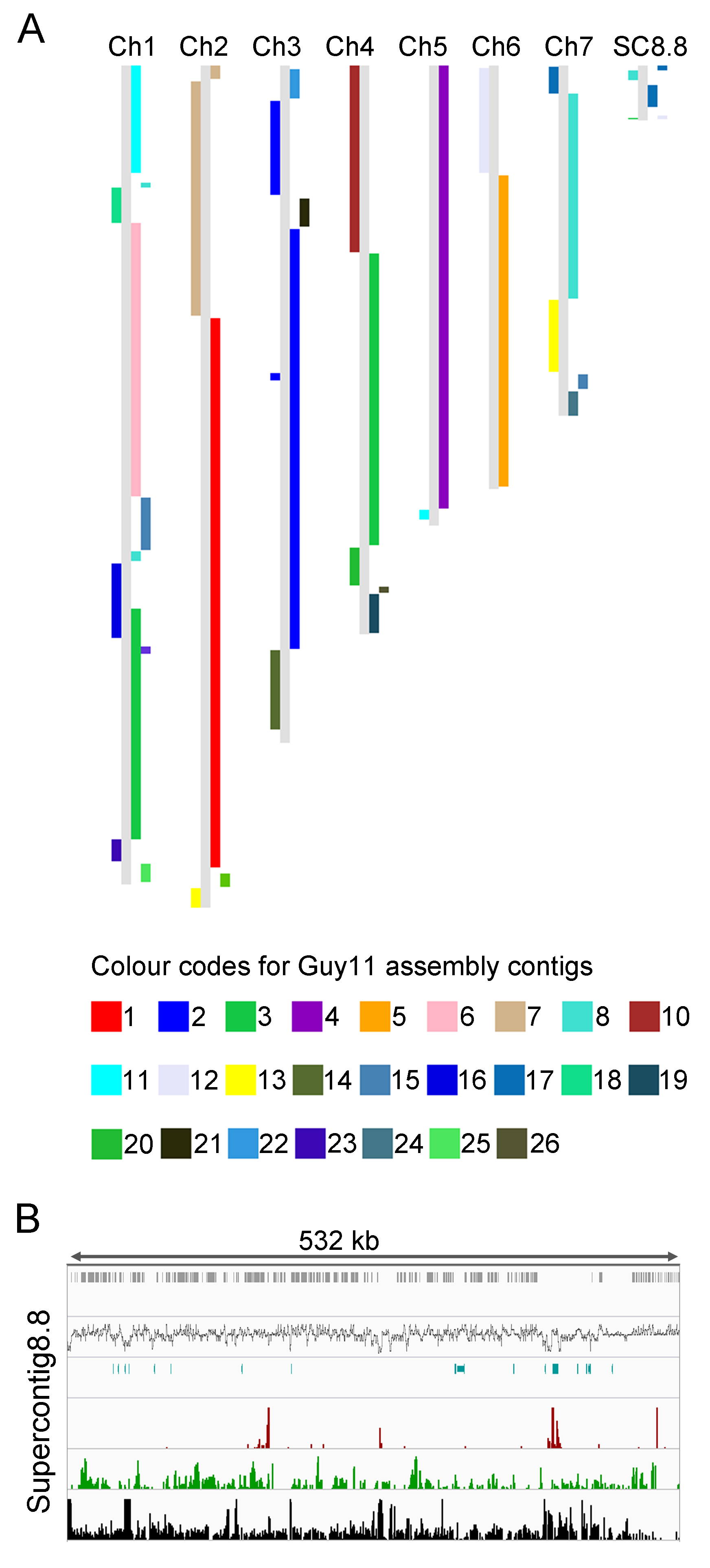

Supplement: FIG S5 [file mBio.01581-19-sf005.tif]

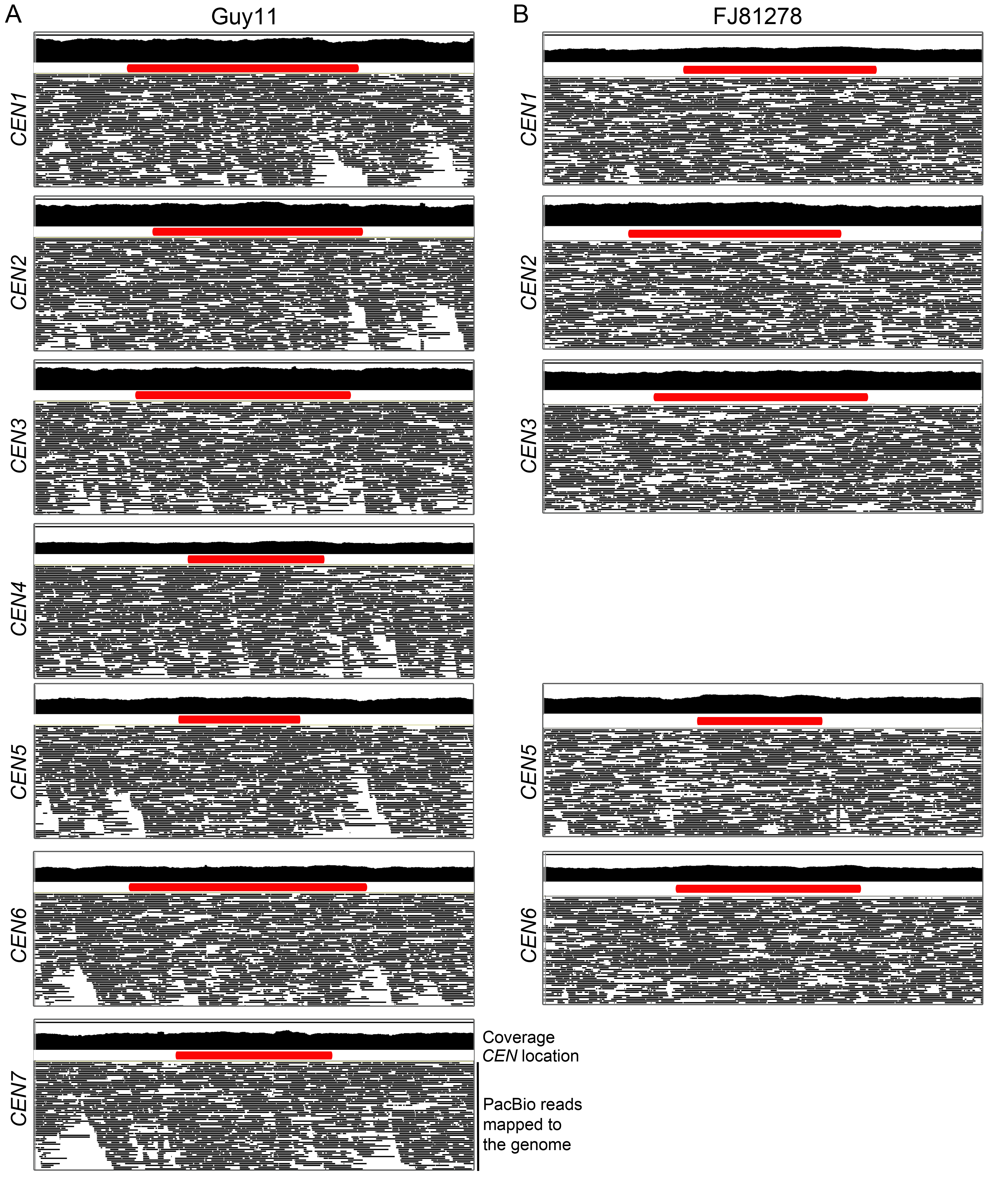

Supplement: FIG S6 [file mBio.01581-19-sf006.tif]
