# Supplementary material for: Cellular Dynamics and Genomic Identity of Centromeres in Cereal Blast Fungus
Source: mBio. 2019 Jul 30;10(4):e01581-19. doi: 10.1128/mBio.01581-19 (PMC6667624; doi:10.1128/mBio.01581-19)
Supplement: TEXT S1 [file mBio.01581-19-s0001.docx]

**Text S1 (Supplementary Methods)**

**Plasmid constructs for epifluorescent marker-tagged strains of *M. oryzae*.**

**Histone H1-mCherry (nuclear marker):** The PCR amplified mCherry open reading frame (ORF), without the start codon, was cloned into the plasmid vector pFGL822 (Addgene #58225), which contains the Basta (Phosphinothricin/Glufosinate) resistance cassette, using KpnI and BamHI restriction enzymes. Next, histone H1 ORF (without the stop codon) and its 3’UTR sequence were PCR amplified with the corresponding primers (listed in the Table S4) and ligated sequentially to generate the final plasmid pFGL1170R (Addgene #116896).

**CenpC-GFP and CenpC-mCherry (kinetochore markers):** The GFP ORF (without the start codon) was introduced into plasmid pFGL821 (Addgene #58223), which contains the Hygromycin B resistance gene. The C-terminal part of CenpC ORF (without the stop codon) and 1 kb of 3’ UTR were subsequently cloned into the plasmid to obtain CenpC-GFP reporter plasmid pFGL1079 (Addgene #116897). CenpC-mCherry construct pFGL1169R (Addgene #116899) was generated in a similar fashion to that of hH1-mCherry by cloning the CenpC ORF and the 3’ UTR.

**GFP-CenpA (kinetochore marker):** A codon-optimized Tet-off regulator cassette was commercially synthesized and cloned into the backbone plasmid pFGL1252 (Addgene #118991). Subsequently, a hygromycin resistance cassette was ligated in pFGL1252 to obtain the Tet-off plasmid pFGL1252_TetOFF (Hyg) (Addgene #118992). The GFP ORF (without the stop codon) was introduced immediately after the modified Tet-off cassette to obtain pFGL1252_TetGFP (Hyg) (Addgene #118993). The entire CenpA ORF (lacking the start codon) along with its 3’UTR and 1 kb of 5’ homology arm was cloned and ligated sequentially into pFGL1252_TetGFP(Hyg) to generate the Tet-off controlled GFP-CenpA expressing plasmid pFGL1258 (Addgene #116898).

**GFP-TubA and mCherry-TubA (microtubule marker):** The TubA ORF (without the ATG start codon) with its 3’UTR was PCR amplified and cloned immediately after the *GFP* in pFGL1259 (Addgene #118996). The promoter of TubA was then cloned upstream of the *GFP* coding sequence to get the final plasmid pFGL1260 (Addgene #116900). The mCherry-TubA construct pFGL1260R (Addgene #116901) was generated similarly by cloning the same fragments in pFGL1259R (Addgene #118995).

**Alp6-mCherry (Spindle pole body/MTOC marker):** A marker-fusion reporter construct, pFGL1269R (Addgene #118994), was used as the backbone vector for this plasmid construct. The C-terminal part of Alp6 ORF (without the stop codon) and 1 kb of its 3’ UTR were cloned into requisite sites upstream and downstream of the *mCherry* respectively, yielding the final plasmid pFGL1344 (Addgene #116902).
