# Supplementary material for: Cellular Dynamics and Genomic Identity of Centromeres in Cereal Blast Fungus
Source: mBio. 2019 Jul 30;10(4):e01581-19. doi: 10.1128/mBio.01581-19 (PMC6667624; doi:10.1128/mBio.01581-19)
Supplement: TABLE S1 [file mBio.01581-19-st001.docx]

**Table S1. List of strains, plasmids and primers used in this study.**

1. **List of validated strains used in this study.**

| **Strain** | **Brief description** | **Purpose** | **Selection** |
| --- | --- | --- | --- |
| Guy11 | Wild type (MAT1-2) | Parent stain for fungal transformation | None |
| MGYF01 | Guy11 transformed with pFGL1258 | GFP-CenpA tagging under Tet-off promoter regulation | Hyg |
| MGYF02 | Guy11 transformed with pFGL1079 | CenpC-GFP tagging | Hyg |
| MGYF03 | MGYF01 transformed with pFGL1170R | GFP-CenpA and H1-mCherry | Hyg, Bar |
| MGYF04 | MGYF02 transformed with pFGL1170R | CenpC-GFP and H1-mCherry | Hyg, Bar |
| MGYF05 | MGYF01 transformed with pFGL1169R | GFP-CenpA and CenpC-mCherry | Hyg, Bar |
| MGYF06 | MGYF03 transformed with pFGL1260 | GFP-CenpA, H1-mCherry and GFP-TubA | Hyg, Bar, Sur |
| MGYF07 | MGYF01 transformed with pFGL1260R | GFP-CenpA and mCherry-TubA | Hyg, Sur |
| MGYF08 | MGYF01 transformed with pFGL1344 | GFP-CenpA and Alp6-mCherry | Hyg, Bar |
| MGYF09 | MGYF08 transformed with pFGL1260 | GFP-CenpA, Alp6-mCherry and GFP-TubA | Hyg, Bar, Sur |

1. **Plasmid constructs used in this study.**

| **Constructs**  **(Addgene #)** | **Targeted gene** | **Description/purpose** | **Selection marker** |
| --- | --- | --- | --- |
| pFGL1170R  (116896) | hH1 (MGG_12797) | C-terminal tagging with mCherry at the native locus, as a nuclear marker. | Basta |
| pFG1079  (116897) | CenpC (MGG_06960) | C-terminal tagging with GFP at the native locus, as a kinetochore marker. | Hygromycin |
| pFGL1258  (116898) | CenpA (MGG_06445) | N-terminal tagging with GFP with a Tet-OFF cassette at the native locus, as a kinetochore marker. | Hygromycin |
| pFGL1169R  (116899) | CenpC (MGG_06960) | C-terminal tagging with mCherry at the native locus, as a kinetochore marker. | Basta |
| pFGL1260  (116900) | TubA (MGG_11412) | N-terminal tagging with GFP at the ILV2 locus, as a microtubule marker. | Sulfonylurea |
| pFGL1260R  (116901) | TubA (MGG_11412) | N-terminus tagging with mCherry at the ILV2 locus, as microtubule marker. | Sulfonylurea |
| pFGL1344  (116902) | Alp6 (MGG_01815) | C-terminal tagging with mCherry at its native locus, as an MTOC marker. | Basta |

1. **Oligonucleotide primers used in this study.**

| **Primer Name** | **Sequence (5' to 3')** | **Application** |
| --- | --- | --- |
| GFP_F4_KpnI | CATC**GGTACC**GTGAGCAAGGGCGAGGAGCTGT | GFP without start codon |
| GFP_R720_BamHI | CGC**GGATCC**TTACTTGTACAGCTCGTCCATGC |  |
| GFP_R717_BamHI | CGC**GGATCC**CTTGTACAGCTCGTCCATGCC | GFP without stop codon |
| mCherry_F4_KpnI | CTC**GGTACC**GTGAGCAAGGGCGAGGAGGATAA | mCherry without start codon |
| mCherry_R711_BamHI | CGC**GGATCC**TTACTTGTACAGCTCGTCCATGC |  |
| mCherry_R708_BamHI | CGC**GGATCC**CTTGTACAGCTCGTCCATGCCGC | mCherry without stop codon |
| hH1_F4_XhoI | CGT**CTCGAG**CCTCCCAAGAAGGAAACC | 5’ homologous arm of hH1 (ORF without stop codon) |
| hH1_R1039_EcoRI | GTC**GAATTC**TGCGGCGGGTGCCTCGGC |  |
| hH1_endF_PstI | CCG**CTGCAG**TAAAGGGACGCTGACGAACTT | 3’ homologous arm of hH1 |
| hH1_R+871_HindIII | CAT**AAGCTT**CTTTCTTTGACGGGAAAGGGA |  |
| CenpA_F(-804)_EcoRI | GGT**GAATTC**AGTCGCAGGTACATCTCATTA | 5’ homologous arm of CenpA |
| CenpA_R0_EcoRI | GTG**GAATTC**TTTATGTCGGTTTCTATATGGTTTC |  |
| CenpA_F4_BamHI | ATA**GGATCC**CCACCACAAAAAGTAAAGAAGG | 3’ homologous arm of CenpA (ORF+3’ UTR) |
| CenpA_R+425_XbaI | GTT**TCTAGA**AAAGCAGTCCCCAGAGTAACTT |  |
| CenpC_F1704_EcoRI | CCG**GAATTC**GAGGACGAAGAACC | 5’ homologous arm of CenpC (without stop codon) |
| CenpC_R2253_KpnI | GAT**GGTACC**GCTGCTTTCAGTCATTTCGTCC |  |
| CenpC_F2254_PstI | CTG**CTGCAG**TAATTCATCGCGGTGGGTTGGTC | 3’ homologous arm of CenpC |
| CenpC_R+966_HindIII | TGT**AAGCTT**CTGGCCCTCCCTCATTAT |  |
| TubA_F(-1042)_XhoI | GCA**CTCGAG**GCCGCCGGTGTAATTCATGGTGACT | TubA promoter |
| TubA_R3_KpnI | CTC**GGTACC**CATTGTGGATTCTAGGCACTTTTCTCAG |  |
| TubA_F4_BamHI | TCC**GGATCC**AAAGGCGAGGTAGGTGATGCTT | TubA (ORF+3’ UTR) |
| TubA_R+559_XbaI | GCGC**TCTAGA**GGGTGAAACCAAGTATGT |  |
| Alp6_F1876_EcoRI | CTC**GAATTC**GCAATATGCAAGCCCAGAAGTGC | 5’ homologous arm of Alp6 (without stop codon) |
| Alp6_R2876_KpnI | AAC**GGTACC**GTCTCCAGCACGCTCGCCCCTG |  |
| Alp6_endF_XbaI | TGC**TCTAGA**TAAGTAGTTTGAGAAGGAATGACGC | 3’ homologous arm of Alp6 |
| Alp6_R+967_HindIII | ATG**AAGCTT**GGGTCGATGAGTGGTTGGGTT |  |
| HR_F 15.1 | CGCCGTATATTTTCTTCCG | *CEN1* ChIP qPCR |
| HR_R 15.1 | CGAAAATTTCGAAATTCTGCC |  |
| HR_F 7.2 | GACCAAACCTCCTATTACC | *CEN2* ChIP qPCR |
| HR_R 7.2 | GTAAAGGGGAATATTGCCG |  |
| HR_F 2.2 | TGCGCTAAAGATTCCGGG | *CEN3* ChIP qPCR |
| HR_R 2.2 | TCGTTGGTTATATTAAGCGG |  |
| HR_F 10.1 | GGCACCGGAAACCTTTTC | *CEN4* ChIP qPCR |
| HR_R 10.1 | ACGTTGTACGCAATTGGAC |  |
| HR_F 4.1 | CGCACAAAGATTATAAGGTAG | *CEN5* ChIP qPCR |
| HR_R 4.1 | CTCAAATACGTTATTTTAAACAG |  |
| HR_F 5.1 | TTTAACGTCGATTACCATTTC | *CEN6* ChIP qPCR |
| HR_R 5.1 | TAAGACAAATGGGTTCAAATC |  |
| HR_F 13.1 | CGATAAAAACGCGTTTGCG | *CEN7* ChIP qPCR |
| HR_R 13.1 | GAATTTCGTCGGGGTTTTC |  |
| Mg.CEN6.ctrl.FP | CTTTTGCTCAAGAAGCAGCG | Non-*CEN* ChIP qPCR |
| Mg.CEN6.ctrl.RP | TCGGTGTGCGTTTCGTGCTG |  |
| HR_F_62 | GAGTTTATCTCCCGGCTTG | MGG_01054 ChIP qPCR |
| HR_R_63 | CAGAGTACGGATCGCCTC |  |

Note: The restriction enzyme sites are underlined.
